# Explaining Driver Behavior in Sim Racing with Shannon Entropy and LLM Feedback

**DOI:** 10.3390/e28090960

**Published:** 2026-08-27

**Authors:** Tomaz Nunes, Morsinaldo Medeiros, Marianne Silva, João Carlos N. Bittencourt, Daniel G. Costa, Ivanovitch Silva

**Affiliations:** 1 Graduate Program in Electrical and Computer Engineering, Federal University of Rio Grande do Norte, Natal 59078-970, Brazil; tomaz.nunes.098@ufrn.edu.br (T.N.); morsinaldo.medeiros.075@ufrn.edu.br (M.M.); 2 SI, Federal University of Alagoas, Penedo 57200-000, Brazil; marianne.silva@penedo.ufal.br; 3 CETEC, Federal University of Recôncavo da Bahia, Cruz das Almas 44380-000, Brazil; joaocarlos@ufrb.edu.br; 4 SYSTEC, ARISE, Faculty of Engineering, University of Porto, Porto 4200-465, Portugal

**Keywords:** embedded systems, driver behavior permutation entropy, Jensen–Shannon divergence, Edge AI

## Abstract

In some scenarios, motorsport simulators have been used to enable the controlled acquisition of dense telemetry with high similarity to real-world data, reducing cost when assessing driving performance. However, although popular, performance analyses traditionally treat human control as deterministic and overlook the stochasticity of driving behavior. In fact, existing coaching methods which improve driving performance have to deal with two distinct outcomes: a driver who restructures his race control strategy and a driver who merely repeats it faster. This article presents a Behavior-First framework for interpretable driver behavior analysis that separates them. We characterize control signals with two information-theoretic descriptors: Jensen–Shannon divergence, which quantifies distributional distance from a proficiency-matched reference and whose square root satisfies the triangle inequality, and Permutation Entropy to measure the ordinal complexity of the input sequence. A deterministic, physics-informed heuristic layer then identifies kinematic performance gaps and emits structured tokens that a Large Language Model translates into natural-language coaching narratives. We evaluated the framework in an exploratory case study. The three beginners who received generated coaching messages and the single uncoached comparison participant exhibited different lap-time and information-theoretic trajectories. Because the groups were small and non-randomized, these observations describe within-driver evolution and do not estimate a causal coaching effect.

## 1. Introduction

High-fidelity driving simulators have become experimental platforms for studying human driving behavior under controlled, repeatable conditions [1,2,3]. Modern sim racing environments, such as iRacing™ [4], provide realistic vehicle dynamics, laser-scanned circuits, and high-frequency telemetry. This data density enables investigations that would be costly, unsafe, or difficult to replicate in real-world motorsport settings. As a result, sim racing has become a useful testbed for analyzing driver control strategies, behavioral variability, and performance outcomes [5,6,7,8].

The growing availability of telemetry has motivated the development of data-driven coaching and performance analysis methods [9,10]. Using this data, many approaches have focused on lap-time prediction, skill classification, expert-reference comparison, or performance-gap estimation [6,7]. Although these methods provide useful quantitative indicators, they often treat driving primarily as an optimization problem. In practice, they can identify what should change to reduce lap time but offer limited insight into how control actions evolve or why specific track sectors impose greater behavioral demands on the driver [11].

Roughly speaking, driving is a dynamic human control process. Steering, braking, and throttle inputs are continuous signals from the driver, shaped by vehicle dynamics, sensory feedback, and cognitive effort [12,13]. In this context, variability in these signals is not necessarily a sign of poor performance, as it may reflect adaptive correction, local physical demands, or exploratory control during learning [14]. Therefore, without explicit behavioral characterization, telemetry-based coaching systems may conflate execution errors, physical instability, and legitimate control complexity [11]. This can lead to feedback that is technically plausible yet difficult to interpret or insufficiently aligned with the driver’s actual behavior [15].

Recent studies have begun to incorporate predictive models, counterfactual analysis, explainable machine learning, and Large Language Models (LLMs) into telemetry-based coaching pipelines [16]. These approaches are important because they can transform complex telemetry outputs into more accessible recommendations [15]. However, they still tend to prioritize performance outcomes or expert-reference optimization, often without explicitly validating the statistical and temporal structure of driver behavior. In addition, when LLMs are used, the boundary between analytical inference and semantic communication is not always clear, increasing the risk of domain-specific hallucinations [17].

To address this gap, this article proposes a Behavior-First Telemetry framework for interpretable sim racing coaching. The objective is to analyze driver behavior before generating coaching feedback. To that end, the framework first extracts and synchronizes raw telemetry, then combines an exploratory stationarity assessment with information-theoretic descriptors of driver behavior. The stationarity component characterizes signal regularity under the adopted acquisition protocol and is not used as a confirmatory test of driver proficiency or coaching effectiveness. Next, a physics-informed heuristic layer converts telemetry patterns into symbolic diagnostic tokens. Finally, an optimized LLM translates these deterministic rule-based diagnostics into natural-language feedback. In this architecture, the LLM does not perform telemetry inference, optimization, or decision-making, serving only as a semantic interface.

The proposed framework was evaluated through an empirical case study using telemetry sampled at 60 Hz in iRacing. The study involved six volunteer drivers with different proficiency levels: a professional race driver and five urban drivers with self-declared driving skills. The intermediate driver was compared against an expert reference, while the beginner drivers were compared against the intermediate reference, using two different vehicle-track configurations. Among the beginner drivers, one of them did not receive coaching feedback and then was considered for the control group. This tiered design supports proficiency-aware analysis and avoids evaluating all drivers against a single unattainable benchmark.

The novelty claimed in this work is architectural rather than algorithmic. We do not introduce a new entropy estimator, divergence measure, heuristic family, or language model. Instead, the contribution lies in the Behavior-First ordering that places stochastic behavioral characterization and deterministic physics-informed diagnosis upstream of a constrained semantic layer, so that the analytical decisions are made by the stochastic and heuristic stages rather than by the language model, which is dedicated to perform output formatting.

The main contributions of this article are outlined as follows:

- The proposal of a novel Behavior-First telemetry framework that analyzes driver inputs and multiple parameters to deliver user-friendly coaching feedback in sim racing scenarios;
- The combination of a stochastic characterization strategy, a physics-informed diagnostic method, and an LLM-based semantic layer that provides driving feedback in natural language, particularly for non-expert drivers;
- An empirical, simulation-based case study of drivers with different skills, demonstrating the framework’s feasibility.

The remainder of this article is organized as follows. Section 2 reviews related work on telemetry-based coaching and interpretable driver modeling. Section 3 presents fundamental concepts. Section 4 describes the proposed framework, including the stochastic characterization, entropy and stationarity analyses, and the interpretable coaching heuristics. Section 5 presents the experimental setup and evaluation protocol. Section 6 discusses the results and practical implications. Finally, conclusions and references are presented.

## 2. Related Works

Telemetry-based analysis of driving behavior has been investigated in both real-world and simulator settings, primarily using machine learning, signal processing, and rule-based coaching systems [14]. Most studies frame driving behavior as a classification, prediction, or optimization problem. Among them, common goals include driver style recognition, risk detection, skill classification, lap-time prediction, and feedback generation. Although these approaches provide useful performance indicators, they often focus on performance outcomes rather than the temporal organization of driver control behavior.

Metrics based on information theory have also been used to quantify uncertainty, workload, and irregularity in driving behavior. A representative example is Steering Wheel Entropy (SWE), introduced by [18]. SWE estimates the unpredictability of steering corrections and has been widely adopted as an indicator of mental workload, fatigue, and degraded anticipatory control [19,20]. Despite its relevance, classical SWE focuses primarily on univariate lateral control. This limits its suitability for high-performance racing scenarios, where driver behavior depends on the coupled modulation of steering, braking, and throttle. In sim racing, competitive driving requires continuous management of the available tire forces across braking, rotation, and acceleration phases [21].

Beyond entropy-based descriptors, information theory learning has explored alternative divergence measures. Ref. [22] proposed a Cauchy–Schwarz divergence information bottleneck for regression, where the divergence acts as a differentiable objective that shapes a learned representation. This framework approaches differently when the divergence is not optimized but reported as a sector-level readout comparing empirical telemetry distributions between proficiency-matched drivers. Therefore, it required a bounded [0–1], symmetric quantity whose magnitude remains comparable across sectors, channels, and tracks, which the Jensen–Shannon divergence provides and its square root strengthens to a true metric [23].

Within simulator-based driving research, telemetry has also been used for skill classification, behavioral benchmarking, and driver coaching. Refs. [6,7] used telemetry from Assetto Corsa Competizione (ACC) to model driver performance using machine learning and clustering approaches. Their studies showed that simulator telemetry can distinguish drivers based on performance patterns. Furthermore, ref. [5] employed clustering, and [24] utilized Deep Reinforcement Learning (DRL) to compare human driving behavior with autonomous driving agents in a professional simulator environment.

Expanding on the pedagogical impact of AI instructors, ref. [15] examined how multimodal explanations affect performance and cognitive load in driving simulators. Their findings align with [25] and our own observations: while AI-driven coaching effectively accelerates the learning curve for novices by directing attention and mitigating uncertainty, its impact on expert performance is less pronounced.

Other studies in active coaching systems have proposed a telemetry-based training system that used deterministic rules to provide real-time auditory suggestions [9]. Although the system reported short-term performance gains, retention declined once feedback was removed. More recently, the work in [16] combined counterfactual analysis, SHAP-based feature importance, and LLMs to generate driver coaching feedback. Their approach estimates performance gaps relative to an expert reference and converts them into natural-language recommendations.

These studies demonstrate the value of telemetry for driver modeling and coaching. However, most existing approaches remain centered on performance outcomes, expert comparisons, or predictive modeling, with limited attention to the stochastic structure of driver inputs before feedback is generated. In addition, when LLMs are used, they are often positioned as part of the coaching inference process itself rather than as constrained semantic interfaces over deterministic diagnostics.

This article differs from existing literature in four key ways. First, it does not treat telemetry as the sole data input. Instead, it models driver inputs and vehicle response as stochastic behavioral signals. Second, unlike steering-centered entropy approaches, it computes Shannon Entropy over the combined acceleration magnitude, enabling analysis of both longitudinal and lateral vehicle dynamics. Third, it introduces a physics-informed heuristic layer that converts telemetry patterns into explicit diagnostic tokens before any feedback is generated. Finally, the LLM is not used as a black-box reasoning component. Instead, it acts as a “constrained” semantic interface that translates deterministic rule-based diagnostics into natural-language coaching feedback. We believe these contributions are relevant to this research domain. Table 1 summarizes the reviewed literature.

As shown in Table 1, previous studies mainly emphasized skill classification, behavior benchmarking, rule-based training, or expert-reference coaching. The proposed framework complements these approaches by introducing an intermediate behavioral interpretation layer before feedback generation. This layer provides an exploratory characterization of signal regularity, characterizes behavioral complexity, and grounds the resulting recommendations in explicit heuristics. Consequently, the framework structures the semantic stage around telemetry-derived diagnostic tokens rather than asking the language model to infer driving behavior directly from raw telemetry. However, as an important remark, the degree of traceability achieved by the generated text was not independently evaluated, being framed outside the scope of this study.

## 3. Background

This section presents the physical and stochastic concepts required to support the proposed Behavior-First Telemetry framework. The goal is to establish principles for interpreting telemetry signals in this study as behavioral evidence.

### 3.1. Vehicle Dynamics and Tire Force Limits

In racing conditions, driver inputs are constrained by the tire–road interface. In fact, a vehicle cannot generate unlimited longitudinal and lateral forces at the same time. To model this limitation under combined braking, acceleration, and cornering, the available tire force can be approximated by the friction circle:

(1) $${\left(\frac{F_{x}}{\mu F_{z}}\right)}^{2}+{\left(\frac{F_{y}}{\mu F_{z}}\right)}^{2}\leq 1,$$

where $F_{x}$ and $F_{y}$ denote the longitudinal and lateral tire forces, μ is the tire–road friction coefficient, and $F_{z}$ is the vertical load on the tire [26,27].

**Figure 1:**
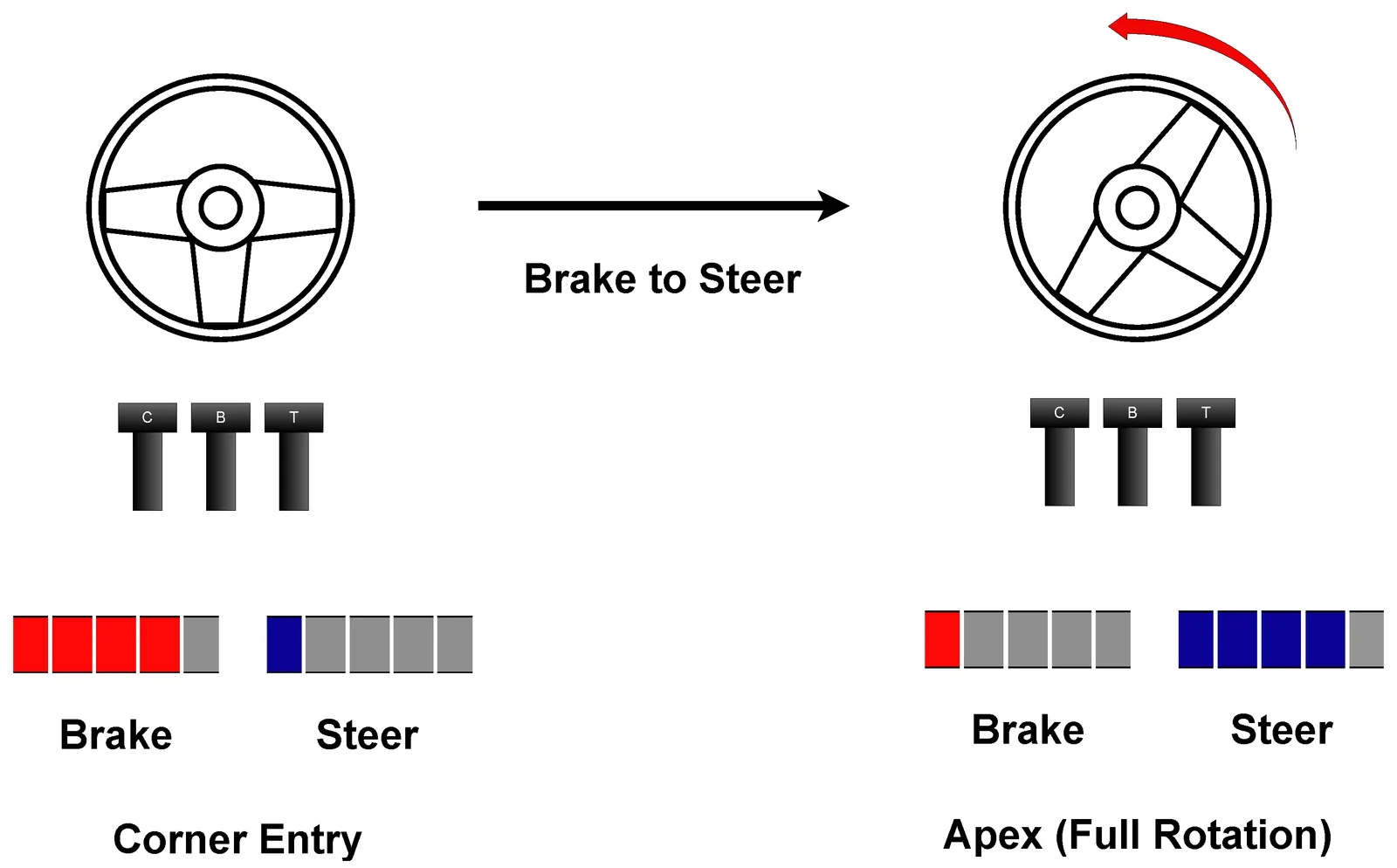
Illustration of the trade-off between brake pressure and steering demand during corner entry.

In this context, an important point should be noted. During corner entry, the driver must progressively trade longitudinal force for lateral force. In practical terms, braking pressure should decrease as steering demand increases. This behavior is commonly associated with trail braking and is often described in coaching literature as a smooth trade-off between brake input and steering angle [21,28]. Figure 1 illustrates this relationship.

From a telemetry perspective, sectors with combined braking and steering tend to produce higher variability in acceleration and control inputs. This variability does not necessarily indicate poor driving; for example, it can reflect structured regulation near the friction limit. For this reason, the proposed framework does not treat signal variability as an error by default. Instead, it evaluates whether variability is consistent, localized, and physically interpretable.

Transient load transfer also affects available grip. As the vehicle accelerates, brakes, or corners, vertical load redistributes across the tires. The longitudinal and lateral load-transfer components can be approximated as:

(2) $$\Delta F_{z,\mathrm{long}}=\frac{h}{L}ma_{x},$$

(3) $$\Delta F_{z,\mathrm{lat}}=\frac{h}{t}ma_{y},$$

where *h* is the center-of-gravity height, *L* is the wheelbase, *t* is the track width, and $a_{x}$ and $a_{y}$ are the longitudinal and lateral acceleration components [26]. These equations explain why braking, steering, and throttle transitions are not independent. Driver inputs continuously reshape the available tire-force budget.

These principles motivate the incorporation of a physics-informed heuristic layer, grounded in vehicle dynamics, into the proposed framework. As a result, the heuristics for braking efficiency, late rotation, steering efficiency, throttle-steering conflict, and inherited exit-speed deficits are grounded in the interaction between tire force limits and load transfer.

### 3.2. Driver Inputs as Stochastic Behavioral Signals

Although vehicle dynamics impose deterministic constraints, driver inputs are not deterministic traces. Steering, throttle, and braking are produced by a human operator who continuously adapts to visual feedback, vehicle response, and perceived grip [28]. For this reason, the proposed framework models telemetry channels as stochastic time series.

In mathematical terms, let X(t) denote a telemetry signal observed over time. For sector-by-sector analysis, it is useful to assess whether the signal behaves as a locally stable stochastic process. A common criterion for this purpose is Wide-Sense Stationarity (WSS). A process is WSS if its mean is constant and its autocorrelation depends only on the time lag:

(4) $$E\left[X\left(t\right)\right]=\mu \;\mathrm{and}\;E\left[X\left(t+\tau \right)X^{*}\left(t\right)\right]=R\left(\tau \right).$$

In this article, stationarity is treated as an exploratory descriptor of local temporal structure within each lap, sector and channel signal rather than as a criterion for accepting or rejecting telemetry signals. The Augmented Dickey–Fuller (ADF) and Kwiatkowski–Phillips–Schmidt–Shin (KPSS) tests are used jointly to examine whether the observed sector-level series are compatible with stationary, non-stationary, or unresolved statistical behavior under the adopted acquisition protocol.

Further, entropy is then used to quantify the degree of behavioral complexity within validated telemetry windows. For a digitized signal *X*, the Shannon entropy is defined as:

(5) $$H\left(X\right)=-\sum_{i=1}^{n}P\left(x_{i}\right)\log_{2}P\left(x_{i}\right),$$

where $P(x_{i})$ is the probability of observing the value $x_{i}$ within the analyzed window [29]. In driver behavior studies, entropy has been used to quantify uncertainty or workload in control actions, particularly through steering measures [18,19,20].

### 3.3. Jensen–Shannon Divergence

Shannon entropy characterizes the behavioral complexity of one driver in isolation. Comparing two drivers requires a measure of distributional divergence. The Kullback–Leibler (KL) divergence between probability distributions *P* and *Q* is:

(6) $$D_{\mathrm{KL}}\left(P∥Q\right)=\sum_{i=1}^{n}P\left(x_{i}\right)\;\log_{2}\frac{P(x_{i})}{Q(x_{i})}.$$

where $x_{i}$ represents the *i*-th possible discrete outcome within the evaluated sample space and *n* is the total number of mutually exclusive events.

KL divergence is asymmetric and unlimited, hence $D_{\mathrm{KL}}\left(P∥Q\right)\neq D_{\mathrm{KL}}\left(Q∥P\right)$, and it diverges when $Q(x_{i})=0$ for any $x_{i}$ in the support of *P*. These properties are undesirable for symmetric driver comparisons.

The Jensen–Shannon Divergence (JSD), introduced by Lin [23], resolves both limitations. Let $M=\frac{1}{2}\left(P+Q\right)$ be the mixture distribution. Then

(7) $$\mathrm{JSD}\left(P\;∥\;Q\right)=\frac{1}{2}\;D_{\mathrm{KL}}\left(P∥M\right)+\frac{1}{2}\;D_{\mathrm{KL}}\left(Q∥M\right).$$

With $\log_{2}$, the JSD is bounded: JSD(P‖Q)∈[0, 1]. A value of 0 indicates identical distributions in a given sector; a value of 1 indicates maximal divergence. Moreover, $\sqrt{JSD}$ satisfies the triangle inequality and is therefore a true metric [30], which supports its use for pairwise driver comparisons.

In this framework, *P* and *Q* are sector-level histograms of the same telemetry channel for the reference and test drivers, computed over a shared fixed partition of n=20 bins. The resulting symmetric divergence map localizes the sectors in which the test driver’s input distributions depart most from the reference, and these sectors become the primary targets for coaching intervention.

### 3.4. Permutation Entropy

Permutation Entropy (PE) quantifies the complexity of a time series from the ordinal structure of its samples rather than their absolute amplitudes. Unlike Shannon entropy computed on static histograms, PE captures the temporal dynamics of human control [31].

The algorithm computes the relative frequency, or probability P(π), of each permutation occurring within the evaluated signal window. Finally, the classical Shannon entropy formula is applied to these probabilities to quantify the temporal complexity:

(8) $$PE\left(m\right)=-\sum_{i=1}^{m!}P\left(\pi_{i}\right)\log_{2}P\left(\pi_{i}\right)$$

The resulting PE value is bounded between 0 and $\log_{2}\left(m!\right)$. A value of zero indicates a perfectly predictable, monotonic sequence (e.g., a driver smoothly and continuously pressing the throttle, generating identical ordinal patterns). On the other hand, higher values indicate greater dynamic uncertainty and a richer structural sequence [31].

We apply PE with embedding dimension m=3 and delay τ=1, yielding 3!=6 possible patterns. A configuration that captures micro-structural variations in driver inputs at the 60 Hz sampling rate. We define the differential $\Delta PE=PE_{n}^{\mathrm{test}}-PE_{n}^{\mathrm{ref}}$, so that positive values indicate that the test driver exhibits more disordered control than the reference in that sector.

### 3.5. Behavioral Complexity and Coaching Interpretation

Entropy should not be taken as a direct measure of driving skill. High entropy may indicate poor control, but it may also reflect structured behavior near the physical limit. Likewise, low entropy may indicate smooth, expert control, but it may also reflect conservative under-driving [20]. Its interpretation depends on the track sector, vehicle state, and driver proficiency.

For this reason, the proposed framework distinguishes between behavioral complexity and diagnostic meaning. Entropy indicates how variable the control pattern is. The physics-informed heuristic layer then explains why that variation occurs. This distinction is important because the same entropy value may correspond to different behaviors depending on the driving context.

In this article, both functional and exploratory complexity are particularly relevant interpretations for this study. Functional complexity arises when variability increases in technically demanding sectors, such as trail-braking zones, chicanes, or long rotation phases. Exploratory complexity arises when variability reflects a driver still searching for stable control strategies. Conversely, very low variability in a novice driver may indicate subdrive behavior, in which the driver avoids the vehicle’s limits rather than controlling it.

Finally, the described theoretical foundation will support the Behavior-First perspective adopted in the proposed framework, summarized as follows:

1. Lap-time delta identifies where performance is lost;
2. Stochastic and entropy descriptors characterize how the driver behaves;
3. Physics-informed heuristics rule the vehicle dynamics;
4. The LLM layer translates telemetry diagnostics into user-understandable coaching guidance.

## 4. Proposed Approach

The proposed approach is a reproducible framework for analyzing driver behavior in high-fidelity sim racing environments, following a Behavior-First perspective, using stochastic methodology. In contrast to approaches that start with lap-time loss alone, it first examines how the driver controls the vehicle over time and how this behavior relates to the physical demands of each track sector.

This perspective is motivated by the fact that the same lap-time deficit may stem from different causes. A driver may lose time due to late braking, poor rotation, excessive steering, inefficient throttle application, or a speed deficit carried over from the previous corner. To address this, the proposed framework combines telemetry extraction and signal synchronization with stochastic behavioral characterization, physics-informed diagnostics, and semantic feedback to generate interpretable coaching guidance.

**Figure 2:**
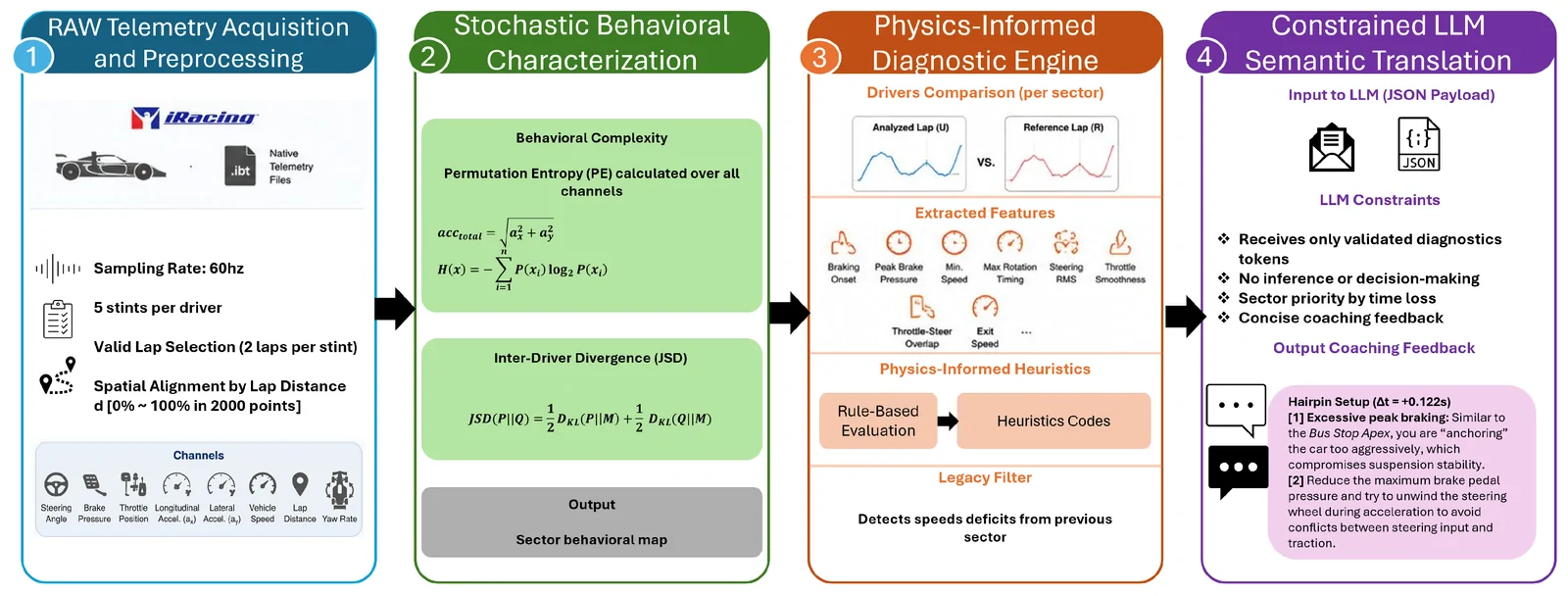
Overview of the proposed Behavior-First Telemetry framework. Raw telemetry is transformed into stochastic descriptors, interpreted using physics-informed heuristics, and converted into natural-language coaching feedback.

Figure 2 outlines the framework’s analytical pipeline. Raw telemetry is first extracted from simulator logs and aligned by lap distance. The synchronized signals are then treated as stochastic time series to assess local regularity and behavioral complexity. Next, physics-informed heuristics compare the analyzed lap with a reference lap and generate symbolic diagnostic tokens. Finally, an LLM converts these activated diagnostic tokens into concise natural-language coaching feedback.

### 4.1. Telemetry Acquisition, Preprocessing, and Stochastic Characterization

Telemetry data is obtained from native iRacing binary telemetry files sampled at 60 Hz. This stage corresponds to the raw telemetry acquisition block shown in Figure 2. Offline extraction was used instead of real-time UDP streaming to preserve signal continuity and avoid network latency or packet loss. This decision is important for stochastic analysis, since stationarity tests and entropy estimation depend on stable signal distributions.

After extraction, the telemetry signals are processed and spatially aligned. For each recorded stint, the parser identifies valid laps and normalizes them to a percentage of lap distance, denoted as d∈[0,100%]. This procedure corresponds to the preprocessing and alignment stage illustrated in Figure 2. It enables laps with different total durations to be compared at equivalent positions on the track. The same spatial reference is later used for stochastic analysis by sector, entropy mapping, and reference-lap comparison.

We select eight telemetry channels: steering angle, brake pressure, throttle position, yaw rate, longitudinal acceleration, lateral acceleration, vehicle speed, and lap distance. Steering, throttle, and brake pressure capture the driver’s control actions while speed and acceleration capture the vehicle’s response to track geometry and tire force limits. Each circuit is further divided into analytical sectors aligned with its corner sequence, enabling sector-level behavioral analysis.

In the proposed framework, these channels are not treated as purely deterministic traces. Instead, we model them as stochastic time series generated by repeated human control actions. This assumption is appropriate because racing inputs are affected by tire saturation, transient load transfer, vehicle balance, and local driver adaptation. Consequently, local signal variability may reflect meaningful behavioral adjustment rather than telemetry noise.

Each experimental stint contains only two valid target laps, so a lap-indexed sequence built from per-lap sector statistics is too short for reliable stationarity inference. Instead of pooling such sequences across stints, the stationarity component takes for analysis the original 60 Hz telemetry segment associated with a single (lap, sector, channel) combination, with approximately 300–1200 samples each track sector. ADF and KPSS are therefore applied directly to the temporal samples recorded while the driver traverses each analytical sector, with no pooling, demeaning, or concatenation across laps or stints. Warm-up and reference stints are excluded from this analysis.

Furthermore, the analysis is performed on the same four telemetry channels used in the information-theoretic characterization: Combined G-Force, Throttle, Brake, and Steering Angle. It has been chosen by us that segments containing fewer than 120 valid samples are not classified, corresponding to a minimum duration of 2 s at the 60 Hz acquisition rate. Near-constant segments, defined by a standard deviation below $10^{-6}$, are also left unclassified because unit-root tests are not informative for essentially invariant signals. This condition occurs mainly for saturated throttle or inactive brake signals.

ADF and KPSS are evaluated jointly using a constant-plus-trend deterministic specification and a significance level of α=0.05. For ADF, the lag order is selected by the Akaike Information Criterion with a maximum lag of 20 samples. For KPSS, the lag bandwidth is selected automatically. Under this specification, the joint outcomes are reported as Stationary, Unit-Root, Inconclusive, or Conflicting. Stationary denotes an ADF rejection combined with a KPSS non-rejection; Unit-Root denotes an ADF non-rejection combined with a KPSS rejection; Inconclusive occurs when neither test rejects its null hypothesis; and Conflicting occurs when both tests reject. These labels describe compatibility with the corresponding joint-test outcome under the adopted constant-plus-trend specification and are not interpreted as direct measures of driver proficiency or control quality.

Driver-level summaries are descriptive. To account for the dependence among sectors and channels recorded within the same lap, uncertainty in the Stationary share is quantified using a lap-block bootstrap with 5000 replicates. Each bootstrap replicate resamples complete laps, preserving all classifiable sector–channel segments from a selected lap as a single block. No significance test is used to compare stationarity verdict distributions between drivers.

Following the exploratory stationarity characterization, we characterize behavioral complexity through the ordinal structure of each control channel using Permutation Entropy (PE) described in Section 3.4 [31]. Unlike Shannon entropy applied to static histograms, PE does not require the ADF/KPSS outcome as an inclusion criterion and is therefore computed independently of the stationarity diagnostic; therefore, a Unit-Root, Inconclusive, or Conflicting verdict does not remove a segment from the PE or JSD analyses. Moreover, PE is computed independently for four telemetry channels at sector resolution: Combined G-Force, Throttle (%), Brake (%), and Steering Angle (rad). Given a telemetry signal X(t) from each of these channels, PE analyzes the relative order of *m* consecutive samples separated by a delay τ; each window maps to one of m! ordinal patterns π.

At the end of this stage, each stint is represented by a sector behavioral map that includes stationarity labels, entropy values and telemetry descriptors. These outputs correspond to the intermediate analytical products depicted in Figure 2.

### 4.2. Physics-Informed Heuristic Engine

The fourth stage in Figure 2 converts the stochastic and kinematic descriptors into symbolic driving diagnostics. Although the stochastic layer identifies where behavioral complexity occurs, it does not explain the physical cause of performance loss. To address this, the framework includes a deterministic, physics-informed heuristic engine.

The comparison is conducted sector by sector. For each sector, the system extracts kinematic and control features from both the driver under analysis and the reference driver. These features include braking onset distance, peak brake pressure, brake release behavior, minimum corner speed, timing of maximum rotation, steering amplitude, throttle application smoothness, and throttle-steering overlap.

Each sector comprises *n* samples on the resampled lap-distance grid, indexed i=1,…,n, with elapsed time $t_{i}$ (s), speed $v_{i}$ (km/h), brake input $b_{i}$ (%), throttle input $\tau_{i}$ (%), steering-wheel angle $\delta_{i}$ (rad), yaw rate $\omega_{i}$ (rad/s), and longitudinal acceleration $a_{i}$ (m/s^2^). The mean sampling interval is $\Delta t=\bar{t_{i+1}-t_{i}}$. Input rates ${\dot{b}}_{i}$ and ${\dot{\tau }}_{i}$ are obtained by central finite differences and smoothed with a Savitzky–Golay filter (window of at most nine samples, third-order polynomial). Braking and traction descriptors are evaluated only on the active-input sample sets $M_{b}=\left\{\;i:b_{i}>5\%\;\right\}$ and $M_{\tau }=\left\{\;i:\tau_{i}>5\%\;\right\}$; when a set is empty, the corresponding descriptors are zero (e.g., long straight). Table 2 defines every descriptor consumed by the heuristic rules of Table 3; each is computed independently for the user lap (*U*) and the reference lap (*R*).

These descriptors quantify how the driver manages braking, rotation, steering efficiency, and traction. The heuristic rules themselves are derived from established vehicle dynamics and race-engineering references [26,27,28], which formalize the trade-offs between tire force limits, load transfer, and control-input timing described in Section 3.1. Each rule therefore analyzes a driving principle—for example, the progressive brake-to-steer trade-off during trail braking, or the coupling between throttle application and steering load near the traction limit. Each heuristic compares the user’s lap, denoted *U*, with the reference lap *R*. When a rule is triggered, the system generates a symbolic diagnostic token that corresponds to a specific driving behavior.

While the structure of each heuristic follows directly from vehicle dynamics theory, the numerical trigger thresholds presented in Table 3 were calibrated empirically through the authors’ hands-on experience analyzing sim-racing telemetry across proficiency levels, rather than fitted through statistical optimization on labeled data or derived from a closed-form physical model. This calibration reflects practical racing-engineering judgment on the magnitude of deviation between *U* and *R* that corresponds to a behaviorally meaningful performance difference. We report this explicitly as a limitation: the thresholds are context-specific to the tested vehicle-track configurations and should be revisited if the framework is extended to other platforms or car classes.

Table 3 summarizes the key heuristics implemented in the coaching engine. The rules are grouped by their dominant driving function. Braking heuristics identify issues with braking-point selection, excessive brake pressure, and low braking efficiency. Rotation heuristics identify delayed rotation, poor trail braking, and inefficient steering behavior. Traction heuristics detect conflicts between throttle and steering or unstable throttle reapplication. Finally, the legacy heuristic identifies time loss carried over from the previous corner.

The legacy rule is important because not every sector with time loss contains a local execution error. A driver may enter a sector already slower due to a poor exit from the previous corner. In this situation, assigning a new local error would lead to a false diagnosis. The legacy filter mitigates this problem by preserving causal coherence across consecutive sectors.

The output of this stage is a structured diagnostic representation. Each relevant sector receives its measured time loss, activated heuristic codes, and the associated physical interpretation. These symbolic diagnostics constitute the deterministic diagnostic representation for the final feedback stage.

### 4.3. Semantic Feedback Translation

The final stage shown in Figure 2 converts the symbolic diagnostic representation into natural-language coaching feedback. The framework uses an LLM solely as a semantic translation interface, acting as a “mediator.” In doing so, it does not perform telemetry analysis, performance inference, or heuristic selection, which are common in LLM chatbots. Instead, it focuses only on textual messages to drivers, directly extracted from the computed outputs of previous steps.

The model receives a structured JSON payload containing the sector identifier, time delta, activated heuristic codes, physical interpretation, and legacy status, with no raw telemetry signal provided. Under this restriction, the model has no access to continuous numerical data from which it could derive its own telemetry interpretations.

The prompt also constrains the response format. The model must preserve the priority order defined by time loss, generate concise recommendations, and explicitly identify inherited deficits when a legacy flag is present. The generation parameters are fixed to reduce output variability. In this study, a local Gemma3:12b [32] model was run via the Ollama LLM runner [33] with a temperature of 0.1 and a maximum of 500 generated tokens.

With these hyperparameters, the LLM performs only a linguistic transformation, converting deterministic symbolic diagnostics into concise coaching recommendations. By restricting the model input to structured diagnostic tokens, the architecture is designed to keep the generated text tied to the upstream analysis and to limit unsupported telemetry interpretations. Hallucination frequency and traceability gains, however, were not directly evaluated in the present study.

Consequently, the Behavior-First Telemetry framework maintains an explicit separation between the analytical stages and the semantic generation stage. Raw telemetry is transformed into computed descriptors and deterministic heuristic tokens before reaching the LLM, while the language model is used only to express these predefined diagnostics as coaching feedback.

## 5. Experiments and Validation

This study evaluates the proposed Behavior-First Telemetry framework through an empirical case study conducted on the iRacing platform [4]. This simulator was selected as a test environment because it provides high-frequency telemetry, laser-scanned circuits, and physically consistent vehicle dynamics [34], ensuring controlled conditions for driver behavior analysis. During the experiments, driver inputs and vehicle responses are recorded in native iRacing files for later processing. This offline approach preserves raw signal continuity and eliminates packet-loss artifacts, enabling verification of whether the resulting telemetry supports behavior characterization and the provision of interpretable feedback at different proficiency levels.

### 5.1. Experimental Setup

The objects of study were two vehicle-and-track configurations selected to subject the framework to different dynamic demands. Table 4 details the configurations used in the case study.

As shown in Table 4, each vehicle was paired with a specific circuit. This design enabled the framework to be evaluated across two combinations of race vehicles and track geometry.

**Figure 3:**
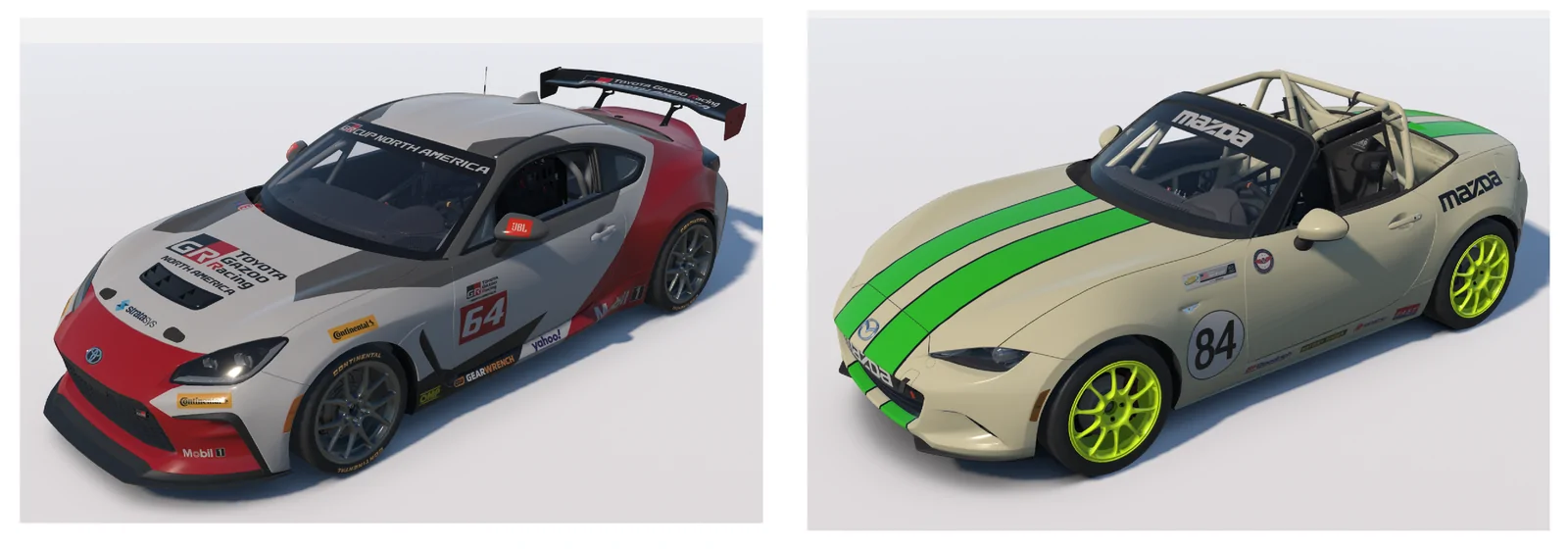
Test vehicles used in the study. The Toyota GR86 Cup is shown on the left and the Mazda MX-5 Global Cup on the right.

The two selected vehicles are illustrated in Figure 3. Both are entry-level sports cars with limited aerodynamic downforce. Their performance depends strongly on mechanical grip, braking stability, load transfer, and throttle modulation, making them suitable for observing driver performance.

The vehicles shown in Figure 3 also exhibit distinct dynamic responses. The Mazda MX-5 Global Cup shows more pronounced pitch and roll, which amplifies load-transfer signatures. The Toyota GR86 Cup offers a different chassis response, enabling better performance comparisons. Regarding the selected race tracks, Charlotte Motor Speedway Roval combines banked oval sections, heavy braking zones, chicanes, and an infield road course. On the other hand, Summit Point Raceway is a road course on natural terrain with variable corner radii and longer periods of sustained lateral load.

**Figure 4:**
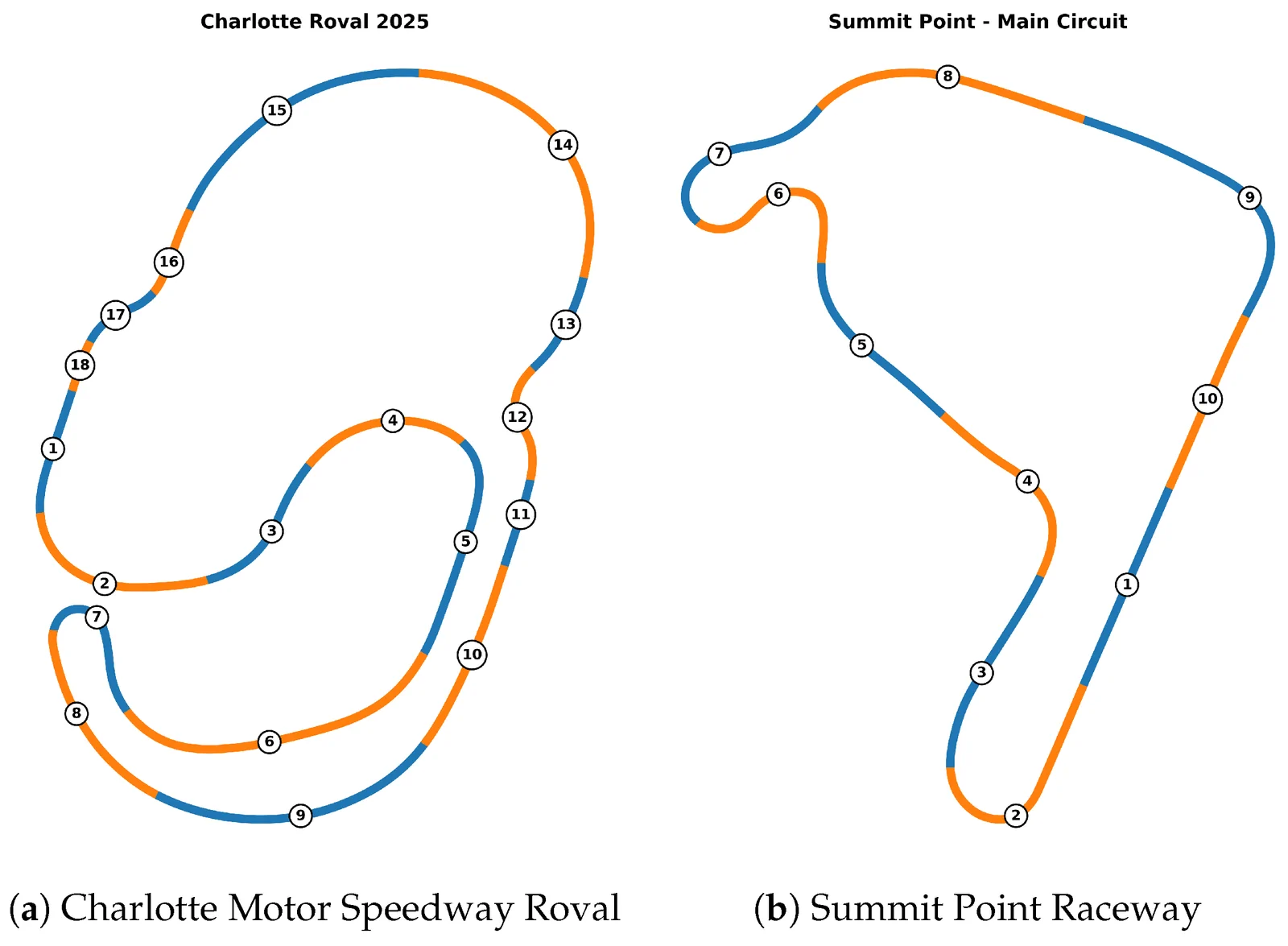
Track layouts discretized into analytical micro-sectors. Charlotte Motor Speedway Roval was divided into 18 sectors, while Summit Point Raceway was divided into 10 sectors.

To support spatially localized analysis, both tracks were divided into analytical micro-sectors. Figure 4 illustrates the sectorization used for each circuit. The Charlotte Roval track was partitioned into a larger number of sectors due to its combination of oval transitions, chicanes, and infield corners, while Summit Point was divided into fewer sectors, each with longer cornering regions that emphasize sustained rotation and throttle control.

The sector labels used throughout the analysis are detailed in Table 5. These labels are used consistently across the time-delta analysis, stationarity tests, entropy mapping, heuristic diagnosis, and semantic feedback generation. In fact, the table shows that sectorization was defined based on each layout’s relevant regions, allowing the analysis to isolate the target variables (i.e., braking zones, corner entry, apex behavior, exit traction, and inherited speed deficits).

Six anonymized drivers were selected to represent different proficiency levels. Driver A represents the expert profile with competitive experience, and is considered the upper performance reference. Driver B represents the intermediate profile, with moderate experience and expected consistent lap execution. Finally, drivers C, D, E and F represent a beginner profile, with limited track knowledge and less stable control behavior.

The case study adopted a tiered differential comparison strategy, in which beginner drivers were compared against Driver B to identify fundamental execution errors, while Driver B is compared against Driver A to identify higher-level technical limitations. This design avoids comparing all drivers against a single unattainable benchmark and makes the feedback compatible with each driver’s learning stage.

All participants completed the driving sessions from their own sim racing setups, using their personal cockpits and individual iRacing accounts. Despite using different hardware, all drivers used load-cell pedals, which naturally produce fast lap times when compared to Hall sensor ones [8]. This approach preserved a naturalistic usage context. After each session, the drivers exported the raw telemetry files and uploaded them to a centralized cloud repository we provided.

### 5.2. Data Collection and Processing

Following the defined pipeline, the data files are received, and relevant telemetry channels are extracted. Here, the objective is to align laps by percentage distance, compute descriptors for each sector, and generate the physics-informed diagnostic tokens.

The selected telemetry channels include steering angle, brake pressure, throttle position, longitudinal acceleration, lateral acceleration, vehicle speed, yaw rate and lap distance. These channels were selected because they jointly capture driver commands and vehicle response. They also support the desired stochastic entropy-based outcome analyses.

Overall, the data collection procedure follows a structured five-stint protocol designed to separate pre-coaching and post-coaching driving behavior across proficiency tiers. The reference driver (Expert) completed a single stint per track configuration, used exclusively to generate the initial coaching feedback for the Intermediate driver; it was not subject to any feedback intervention itself.

The other drivers were allowed a 5 min warm-up before stint validation in order to get used to the track and vehicle dynamics. After this period, they ran two valid laps (without off-track limits) to get their run done.

The Intermediate driver completed five stints per configuration car/track. His first stint served as the baseline and was compared against the Expert’s stint to generate LLM-mediated feedback. This process was repeated for each stint from 1 to 4.

Among the four Amateur drivers, three (feedback treatment group) completed five stints each, receiving feedback once, after each stint from 1 to 4 based on a sector-by-sector comparison with the Intermediate driver’s reference stint. The remaining Amateur driver served as a uncoached comparison condition, completing the same five-stint protocol without receiving any feedback.

The uncoached participant provides a descriptive comparison of lap time and telemetry evolution across repeated stints. As this participant is a single non-randomized case, the comparison cannot isolate the effects of coaching from practice, individual differences, or hardware-related signal characteristics. We therefore use this trajectory as an exploratory reference rather than as a causal control condition.

It is important to mention that lap times for reference stints—Driver A for intermediate comparison and Driver B for amateur analysis—have been run in a solo stint with approximately 20 laps prior to this methodology. The best laps for each driver were chosen to be the reference. Table 6 shows reference stint lap times.

### 5.3. Analysis Protocol

The analysis protocol evaluated the framework from three complementary perspectives. First, time-delta analysis identified where performance losses occurred along the lap. Then, stationarity testing and Permutation Entropy were used to characterize local behavioral regularity and control complexity. Finally, the physics-informed heuristics explained the likely causes of the observed deficits.

During the statistical analysis, sector-wise ΔPE shifts are assessed with Wilcoxon signed-rank tests paired by analytical sector, under Benjamini–Hochberg false-discovery-rate control (q<0.05) within each track. For every test, we report the Hodges–Lehmann pseudo-median (the location estimator associated with the signed-rank statistic), the matched-pairs rank-biserial correlation *r* as the effect size, and 95% bootstrap confidence intervals (bias-corrected and accelerated, 10,000 resamples, fixed seed; percentile fallback where BCa degenerates). When all sectors shift in the same direction, *r* saturates at ±1 and its interval is not estimable by resampling; such cases are flagged and uncertainty is read from the pseudo-median interval instead.

Moreover, lap-time trends across stints are tested per driver with an exact permutation test on Spearman’s ρ (five stints yield 5!=120 permutations, so the smallest attainable *p* is 2/120≈0.017), with Theil–Sen slope estimation and distribution-free confidence intervals, again under FDR control across drivers within each track. Inter-driver variability is quantified by a one-way random-effects variance decomposition: the intraclass correlation ICC(1) gives the fraction of ΔPE variance attributable to differences between beginners, with percentile bootstrap intervals obtained by resampling sectors within drivers.

Sectors are repeated observations within each driver and are also spatially ordered along the circuit. Adjacent sectors may therefore share information because vehicle state and control actions can propagate across sector boundaries. Consequently, the sector-paired Wilcoxon tests and their FDR-adjusted *q*-values are interpreted as within-driver statistical screening measures rather than as independent sector-level or population-level inference. Effect magnitude, direction, and consistency across stints and circuits are considered together with the statistical results.

Besides information theory descriptors, an exploratory stationarity analysis was performed independently of the PE and JSD results on each 60 Hz (lap, sector, channel) telemetry segment. Segments with fewer than 120 valid samples or with near-constant values were left unclassified. Classifiable segments were then evaluated with the joint ADF/KPSS procedure described in Section 4.1, and driver-level verdict compositions were summarized descriptively. Lastly, 95% confidence intervals for the Stationary share were obtained via a 5000-replicate lap-block bootstrap.

This heuristic analysis compared the driver’s performance with the corresponding reference lap. For each sector, the framework extracted braking, rotation, steering, and traction features, which were then evaluated using the deterministic rule set defined earlier in Table 3. The resulting symbolic tokens were then used as input to the semantic feedback layer.

### 5.4. LLM Configuration for Feedback Generation

The diagnostic pipeline and the LLM communicated via a structured JSON payload that included the sector name, time delta, activated heuristic codes, physical interpretation, and legacy status. Before generation, we filtered time-loss events with Δt>0.050 s and sorted sectors by deficit magnitude.

The model configuration was held constant across all feedback generations to standardize the semantic translation procedure. These fixed settings control one source of variation in the generation setup, but run-to-run stability was not quantified in the present study. The following prompt instructed the model to preserve the predefined sector order, distinguish inherited deficits from local execution errors, use the corner names provided in the structured input, and generate concise coaching recommendations based only on the supplied diagnostic information.

*You are a professional SimRacing driving coach.*

*Your task is to analyze time deltas and heuristics to improve performance at the Charlotte Roval 2025 and Summit Point.*

*Priority and Memory Instructions:*

*(1) The JSON data is already sorted by the magnitude of time loss from highest to lowest. Maintain this order.*

*(2) If a sector presents the Legacy category, explain that the time loss is not a current execution error, but a consequence of a poor exit from the previous corner.*

*(3) Use official corner names for geographical context.*

*(4) Be objective and generate a maximum of two bullets per corner.*

*(5) If delta_time_s is negative or zero, write: Execution consistent with the reference.*

*Response format:*

*- ### Corner X: [Name] (Δt = +Y.YYYs)*

*- Technical bullets based on the provided heuristics.*

*- Finish with ### Technical Focus Summary listing the three lap priorities.*

This configuration preserves the separation between analysis and language generation. As a result, the telemetry and heuristic layers make the analytical decisions, leaving the LLM to convert rule-based diagnostic tokens into user-understandable feedback.

Finally, to support reproducibility and future replication studies, the complete analytical pipeline was made publicly available on GitHub: https://github.com/conect2ai/Racing4all (accessed on 15 July 2026).

## 6. Results and Discussion

After the experiments, the collected data was processed and extensively analyzed to determine whether adopting our framework offers potential benefits. Initially, lap-time deltas were used to establish baseline performance gaps and identify where time is lost, followed by an exploratory stationarity analysis of the intra-sector 60 Hz telemetry segments. This analysis characterizes local temporal structure within each sector traversal rather than lap-to-lap behavioral repeatability. Next, Permutation Entropy and the physics-informed heuristics were used to characterize and interpret driver behavior. Finally, the specialized LLM layer was analyzed as a semantic interface for post-session coaching feedback.

### 6.1. Performance Deficits and Exploratory Stationarity Diagnostics

Table 7 summarizes the fastest valid laps used for the baseline comparison. The results indicate that Driver B was close to the expert reference on both circuits, with deficits of +0.983 s at Charlotte Roval and +1.600 s at Summit Point.

**Figure 5:**
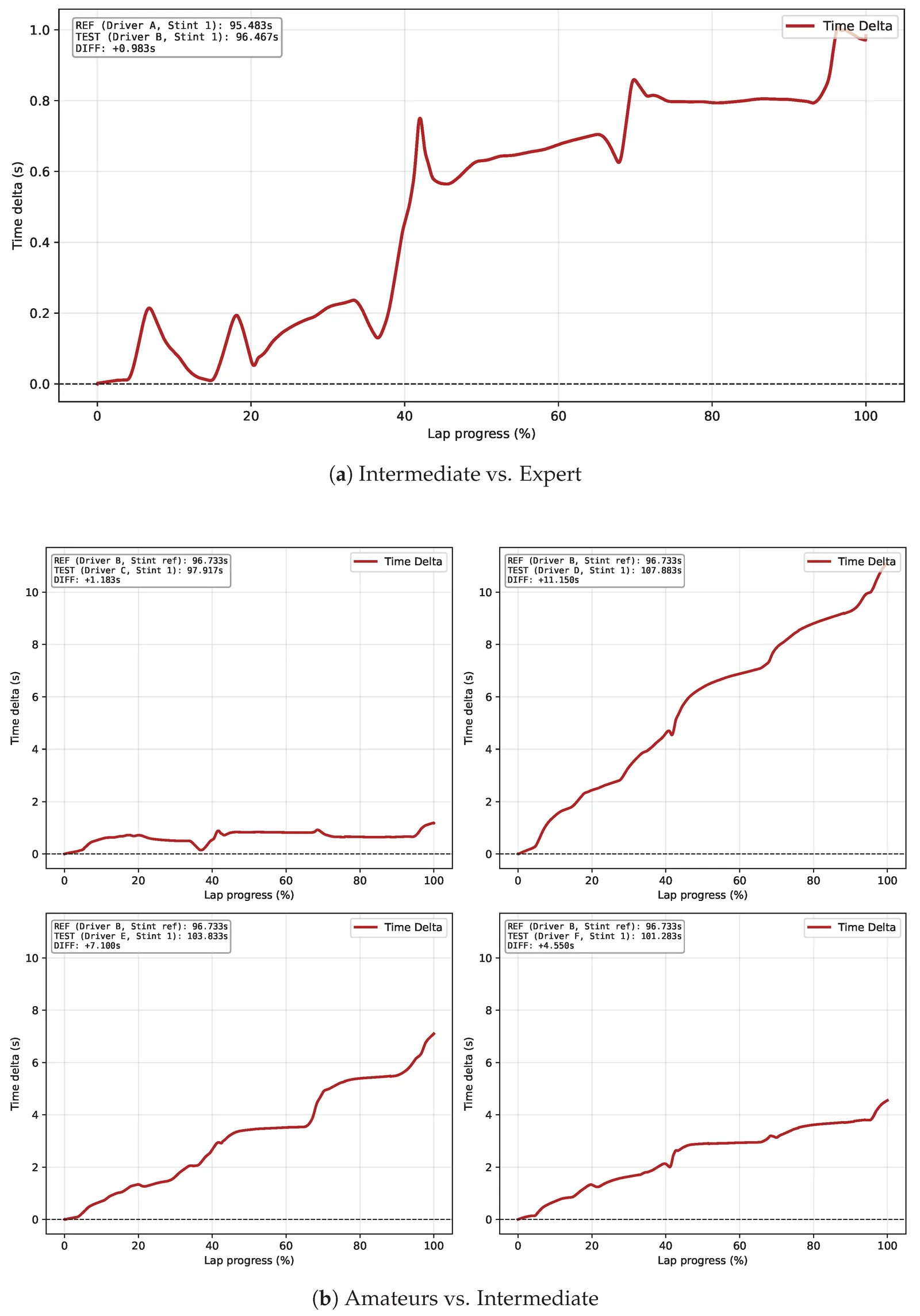
Continuous time-delta analysis over lap progress at Charlotte Roval. Steep upward slopes indicate sectors where the trailing driver loses significant time.

The continuous time-delta profiles provide a more localized view of these deficits, as shown in Figure 5 for the Charlotte Roval track. Driver B loses time to Driver A primarily in localized technical regions, especially near the infield section and the oval transition. His time gap was approximately 1 s behind the experience driver.

Beginner drivers were then compared to intermediate reference. Driver C has a steady delta time loss across all of the Charlotte Roval track, maintaining a time gap of 1.5 s approximately;  Driver D showed the highest gap among all amateurs with a time loss over 11 s; Driver E (who did not receive LLM feedback) had a constant time loss rate until 40% of the circuit (infield), and then had two main losses at the chicanes; and finally,  Driver F had a gap of 4.8 s slower than the reference. The analysis can be seen in Figure 5.

The Charlotte Roval deficits are concentrated in specific high-demand regions. This indicates that the performance gap is not uniformly distributed around the lap. Instead, it arises from localized execution differences in braking, rotation, or corner exit behavior.

**Figure 6:**
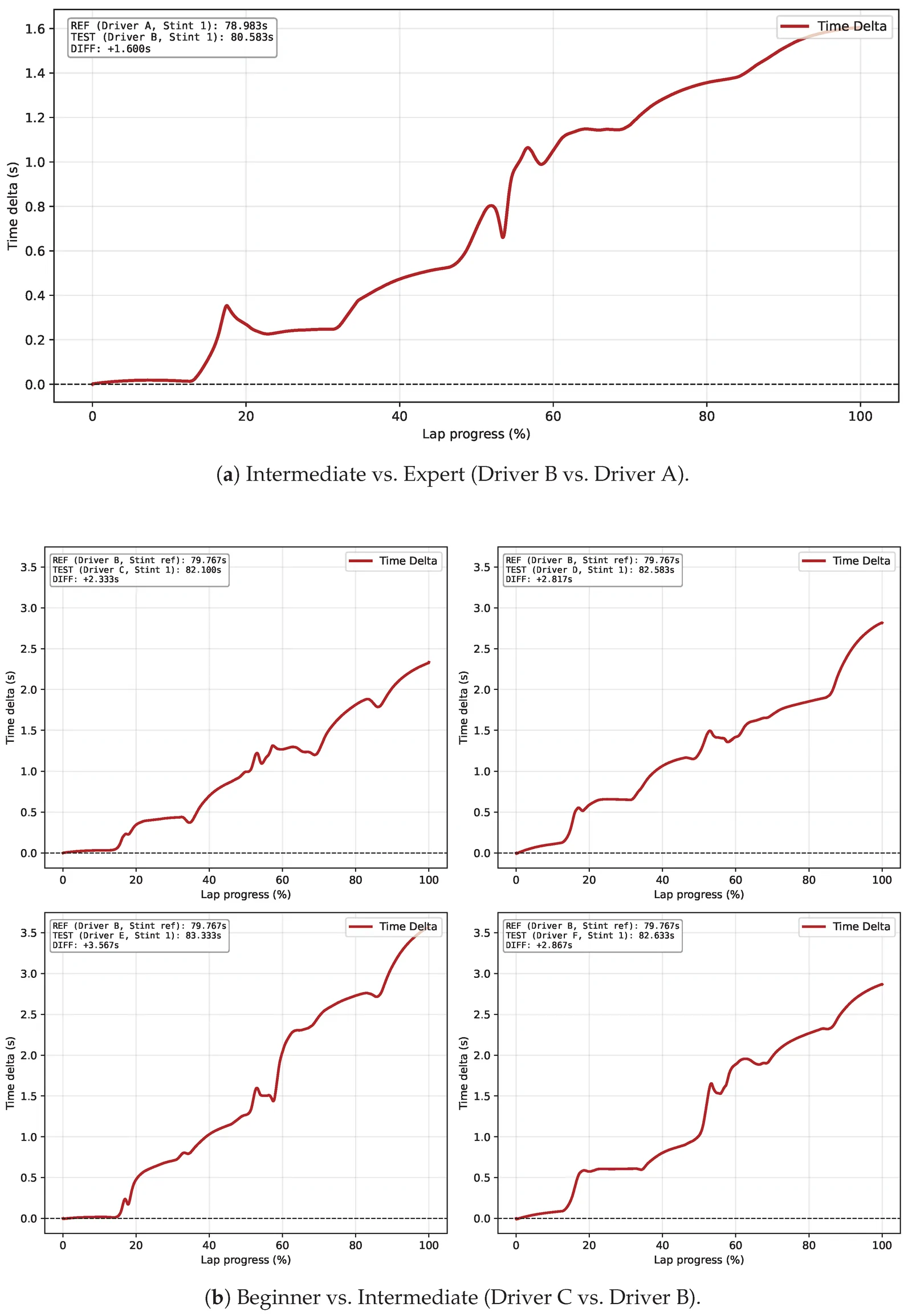
Continuous time-delta profiles at Summit Point. Turn 1 and the Carousel complex emerge as the main deficit region across all beginner drivers.

Figure 6 shows the corresponding time-delta profiles for Summit Point. The Intermediate–Expert comparison shows a progressive deficit accumulation, consistent with a diffuse execution-quality gap across sustained cornering regions. In contrast, the Beginner–Intermediate group comparison shows a more concentrated loss around the Carousel complex and Turn 1, where prolonged trail braking and throttle modulation are required.

Overall, time-delta analysis identifies where performance losses occur but does not characterize the temporal organization of the corresponding control signals. As a first exploratory step toward this dimension, we applied the ADF/KPSS diagnostic directly to the intra-sector 60 Hz telemetry segments, evaluated at α=0.05 with a constant-plus-trend deterministic term.

Across the dataset, 18,296 lap–sector–channel segments were evaluated during our analysis. From this number, 14,466 provided classifiable ADF/KPSS outcomes, whereas 3830 were left unclassified because the segment contained fewer than 120 samples or the signal was near-constant. The latter condition occurred predominantly for Brake and Throttle, for which inactive or saturated inputs naturally produce nearly invariant sequences in some sectors. Table 8 details this breakdown per channel, separating segments excluded by length from those excluded by near-constant signal.

**Figure 7:**
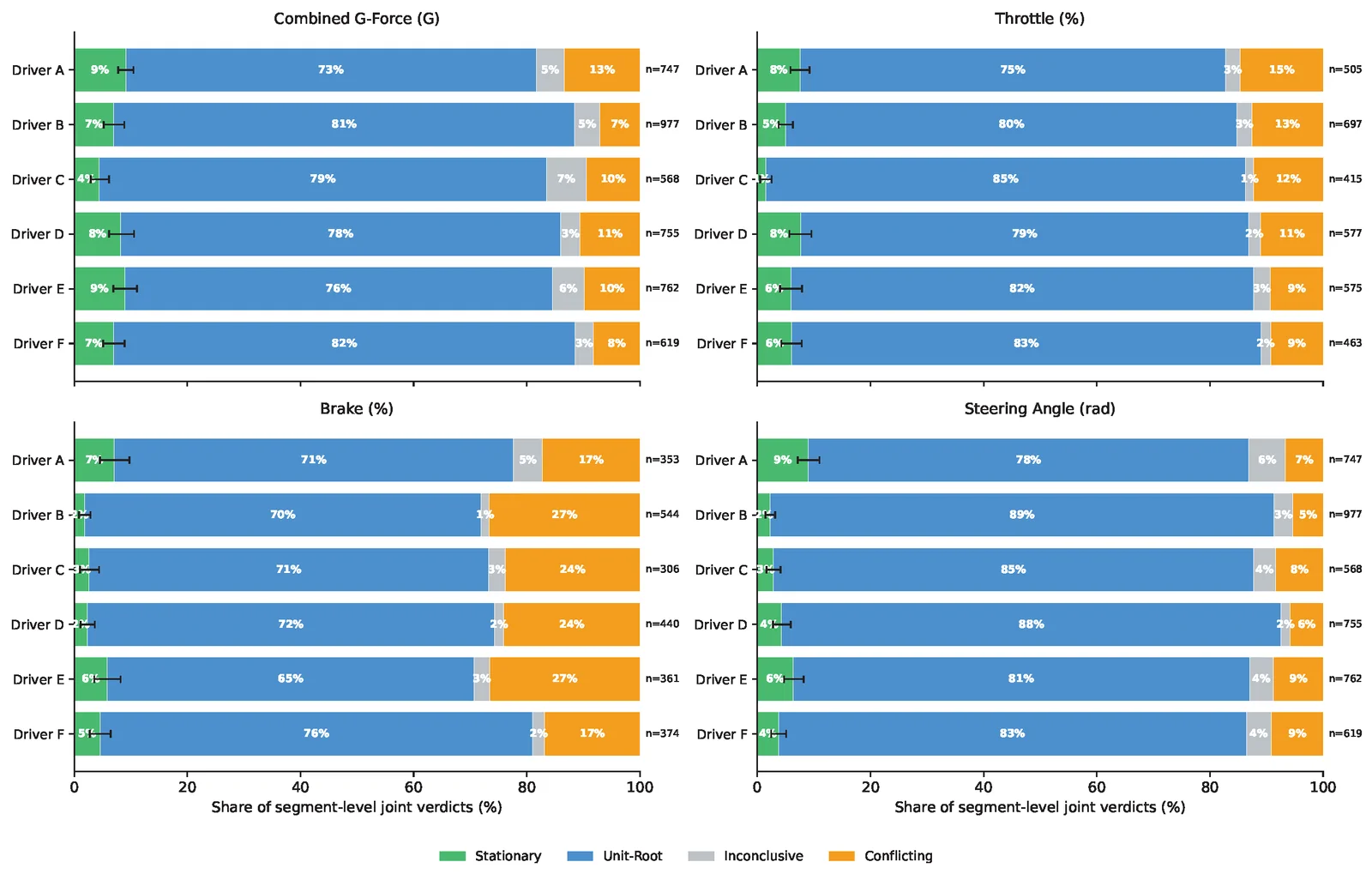
Joint ADF/KPSS diagnostic outcomes for intra-sector 60 Hz telemetry segments, by driver and telemetry channel. Error bars are 95% confidence intervals for the Stationary share (lap-block bootstrap, 5000 replicates); *n* is the number of classifiable segments in each cell.

Figure 7 reports the joint ADF/KPSS outcome of each lap–sector–channel segment under the constant-plus-trend specification, grouped by driver and telemetry channel. These verdicts describe the local temporal structure of each channel and are used neither as indicators of driver proficiency nor as inclusion criteria for the entropy analyses. On those terms, one outcome dominates. Between 65% and 89% of the classifiable segments fall in the Unit-Root category, across every driver and every channel, whereas the Stationary share stays below 10% in all cells.

Differences are more evident across telemetry channels than across proficiency levels. Brake exhibits a comparatively larger proportion of Conflicting outcomes, whereas Steering and Throttle are dominated by Unit-Root outcomes. This behavior is plausible because these control signals evolve systematically during a sector as the driver transitions through braking, rotation, and acceleration phases. Therefore, a Unit-Root outcome is not interpreted as driver instability or poor control; it indicates that the local temporal sequence is not compatible with the Stationary joint-test outcome under the adopted specification.

Finally, the stationarity analysis is used only to characterize the temporal structure of the acquired telemetry. It does not provide evidence of driving proficiency, does not quantify coaching effectiveness, and does not determine whether a segment is included in the PE or JSD analyses.

We therefore do not interpret these differences as evidence that stationarity is associated with driving proficiency. Instead, the results show how the joint ADF/KPSS procedure behaves under the present data-acquisition conditions and highlight the uncertainty introduced by short repeated sequences. In this sense, Figure 7 serves primarily as a feasibility assessment of the stationarity component and as a reference for designing future data collection with more valid laps per stint and a larger number of drivers.

### 6.2. Physics-Informed Diagnostics and Temporal Activation Dynamics

After characterizing stochastic-based behavior, we applied the physics-informed heuristic layer to interpret the kinematic origins of the observed performance gaps. Each heuristic compares the analyzed lap against a reference lap and fires when a physically grounded threshold is exceeded, yielding an interpretable, deterministic activation count. We report these counts along two axes: their distribution across the chained driver comparisons (B vs. A, C vs. B, D vs. B, E vs. B, F vs. B), which reference each driver against the intermediate anchor (Driver B) except for the Expert–Intermediate pair, and their evolution across the five stints (S1–S5).

**Figure 8:**
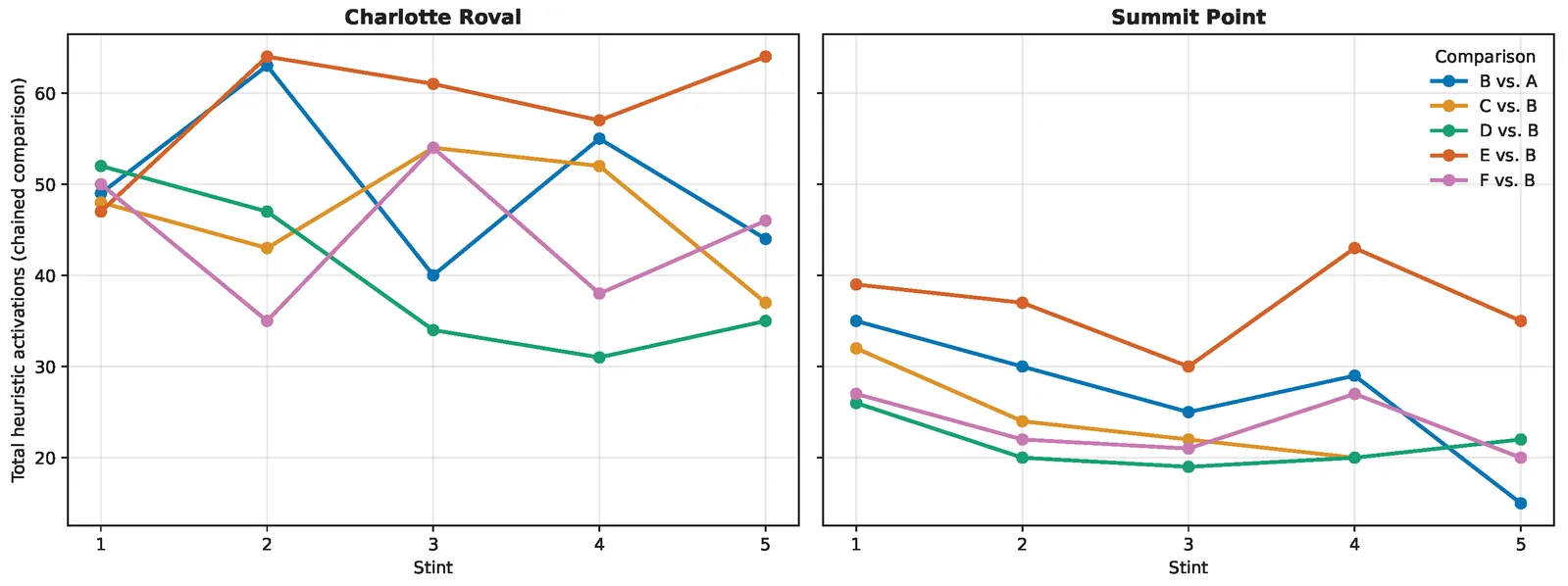
Total physics-informed heuristic activations per chained driver comparison across all stints for Charlotte Roval 2025 and Summit Point.

Beyond this baseline snapshot, Figure 8 tracks the total heuristic activations per comparison across the five stints for both circuits. At Charlotte Roval, the feedback-receiving comparisons trended downward over the session: the Beginner-anchored pair D vs. B declined from 52 activations in S1 to 35 in S5, and F vs. B settled below its S1 level. Summit Point exhibited the same overall descending pattern for the feedback-receiving pairs.

One comparison departed from this trend. The E vs. B pair, in which Driver E belonged to the no-feedback control condition, showed no comparable decline; its activation counts remained high or increased over the session, rising from 47 in S1 to 64 in S5 at Charlotte Roval. We report this contrast as an exploratory observation. Given the single-driver control condition, it should not be read as a population-level causal estimate, but as an intra-subject pattern consistent with the behavioral descriptors tracking session-to-session change.

**Figure 9:**
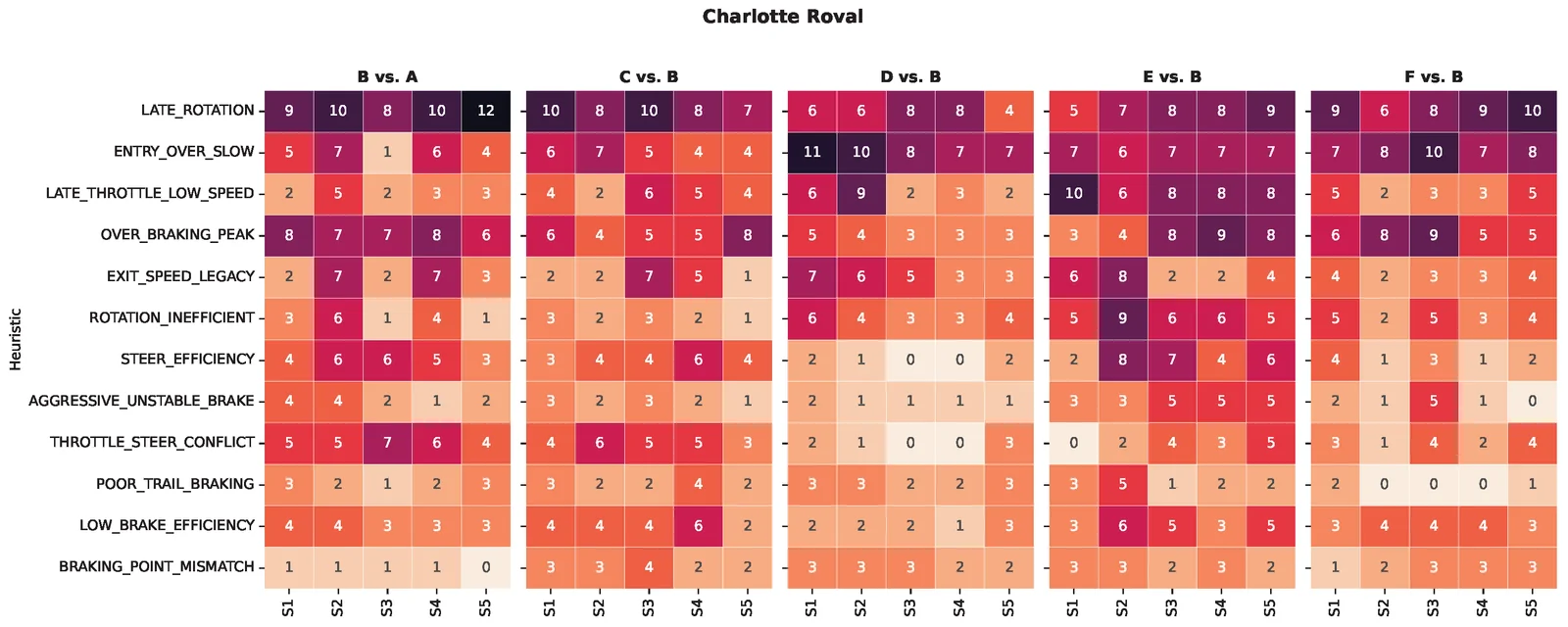
Heuristic activation counts across all stints at Charlotte Roval, for each chained driver comparison.

**Figure 10:**
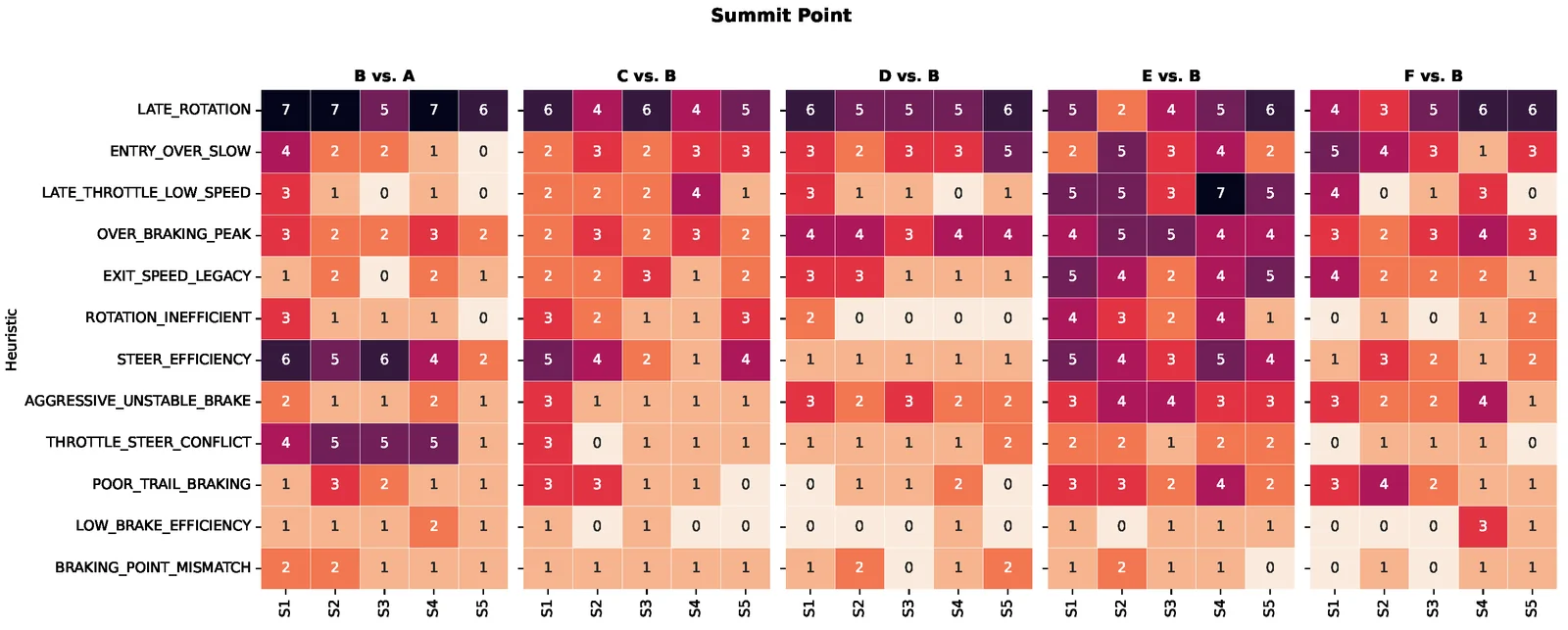
Heuristic activation counts across all stints at Summit Point, for each chained driver comparison.

Figure 9 and Figure 10 resolve this evolution at the heuristic level for Charlotte Roval and Summit Point, respectively. The reduction is not uniform across heuristics: LATE_ROTATION persisted as the dominant trigger through S5, whereas execution-related heuristics such as LATE_THROTTLE_LOW_SPEED and THROTTLE_STEER_CONFLICT decayed more readily.

### 6.3. Information-Structure of Control Behavior

We characterize driver behavior through two complementary information-theoretic descriptors applied to the four primary control channels, notably Brake (%), Combined G-Force, Steering Angle (rad), and Throttle (%) at sector resolution. Jensen–Shannon Divergence (JSD) [23] measures how far apart two drivers’ control distributions are and Permutation Entropy (PE) described by [31] shows how uncertain a single driver’s control sequence is.

We compute JSD with a fixed global binning of n=20 bins per channel and track, which keeps values directly comparable across drivers, sectors, stints, and tracks. JSD is symmetric and bounded in [0,1] for base-2 logarithms [23], and $\sqrt{JSD}$ satisfies the triangle inequality, qualifying as a true metric. We compute PE with embedding dimension m=3 and delay τ=1, normalized per lap and sector. All PE results are reported as $\Delta PE=PE_{\mathrm{test}}-PE_{\mathrm{reference}}$, so positive values indicate greater ordinal complexity in the test driver.

We summarize paired sector-level ΔPE shifts using the Wilcoxon signed-rank test and apply the Benjamini–Hochberg FDR procedure (q<0.05) as a within-driver screening criterion. Because sectors are repeated and spatially dependent observations from the same driver, these *q*-values are not interpreted as evidence of independent sector effects or population-level differences. Instead, they are considered together with effect magnitude, direction, persistence across stints, and recurrence across circuits.

#### 6.3.1. Distributional Divergence Across Stints

**Figure 11:**
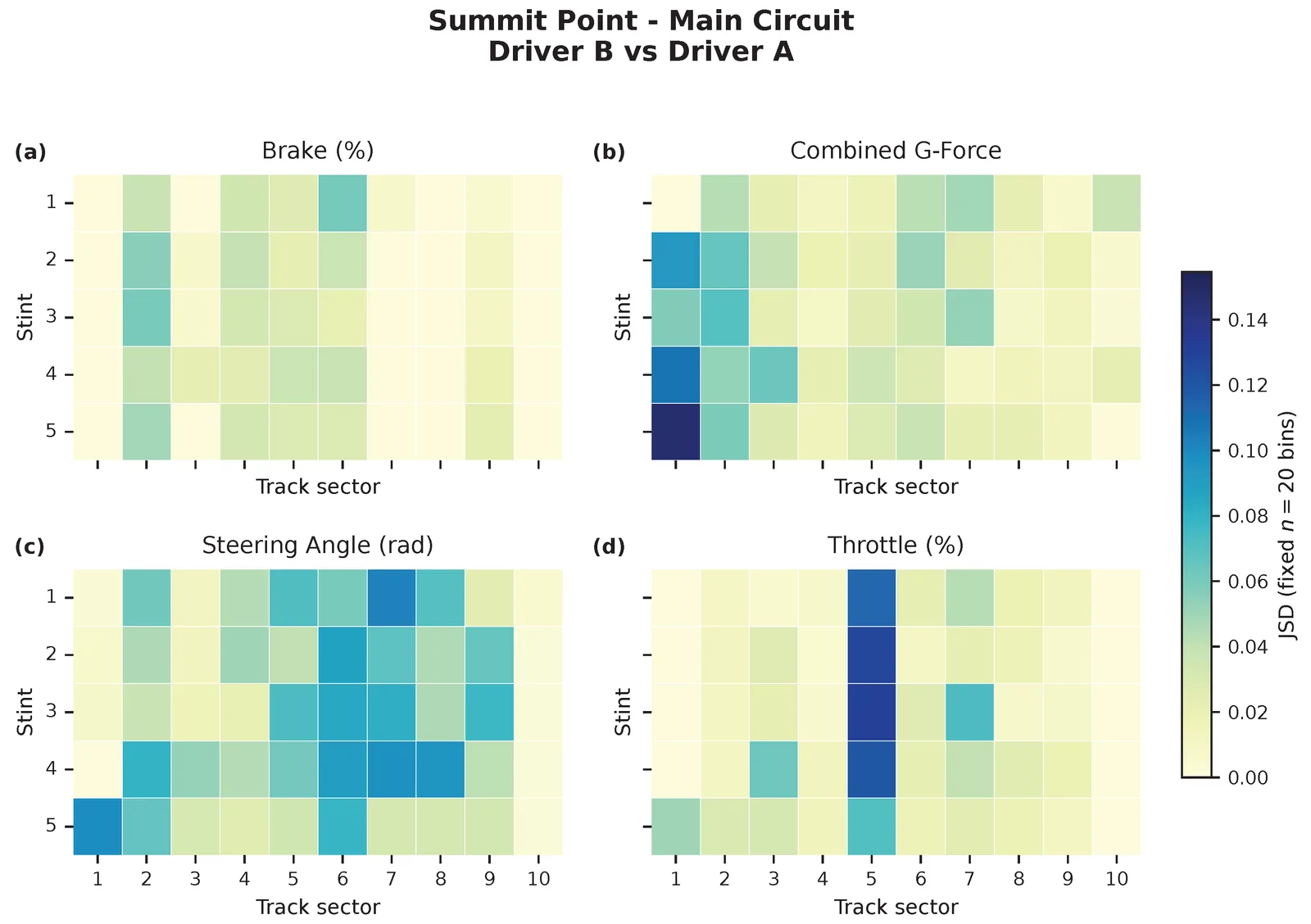
Sector × stint JSD heatmaps for Driver B relative to Driver A at Summit Point.

**Figure 12:**
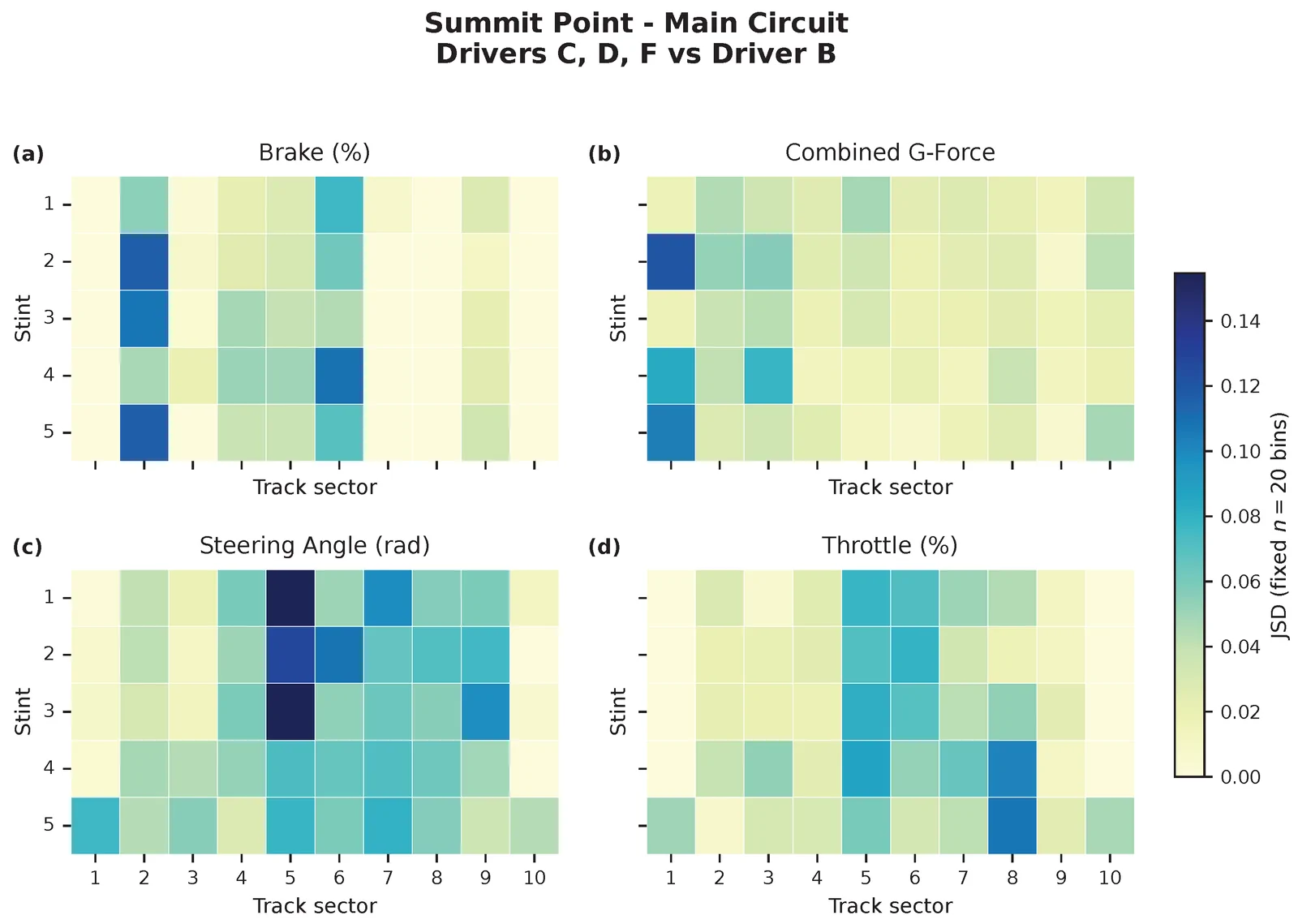
Sector × stint JSD for Drivers C, D and F compared to Driver B at Summit Point.

**Figure 13:**
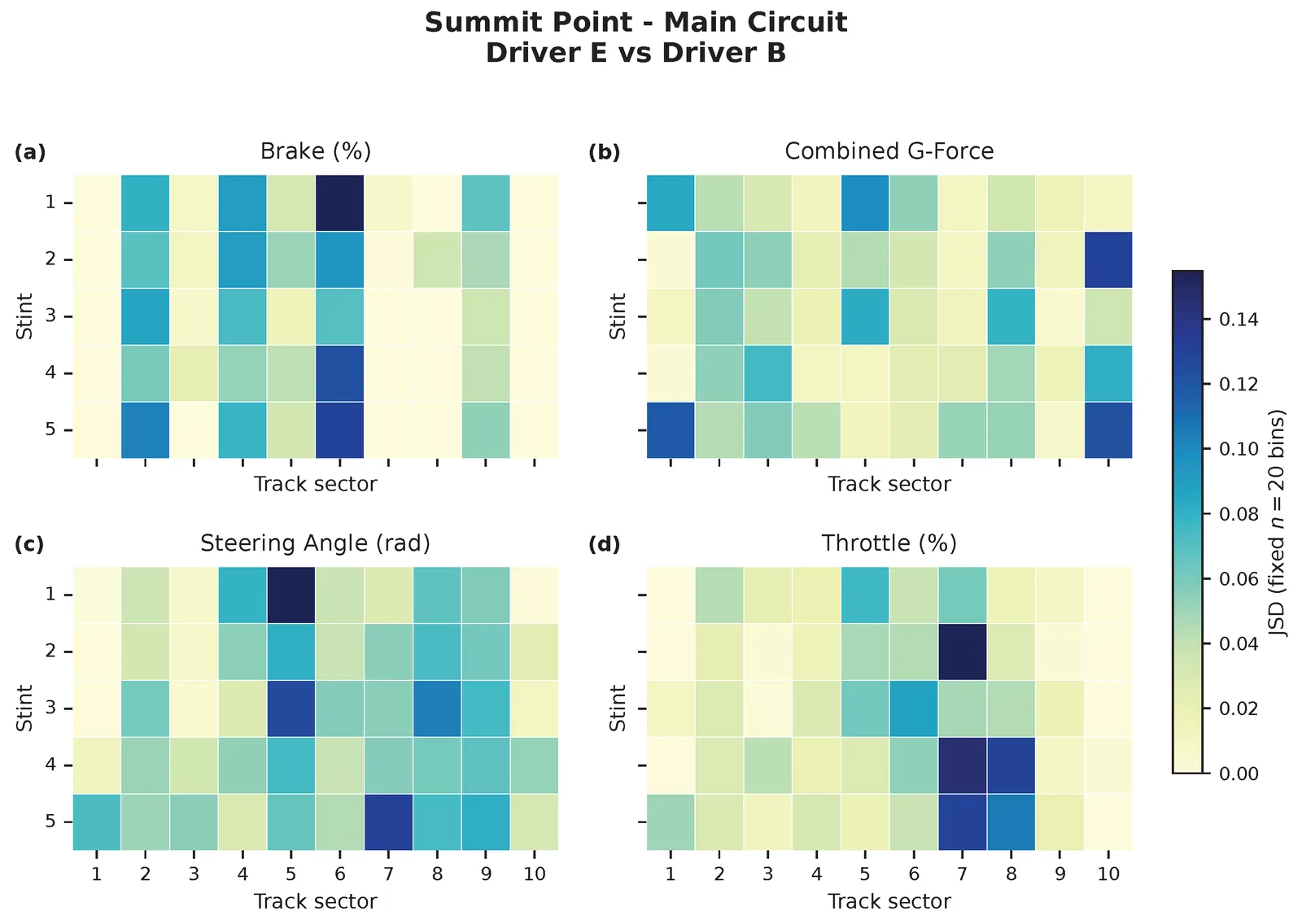
Sector × stint JSD for the uncoached driver (E) relative to Driver B at Summit Point. No coaching feedback was delivered between stints.

**Figure 14:**
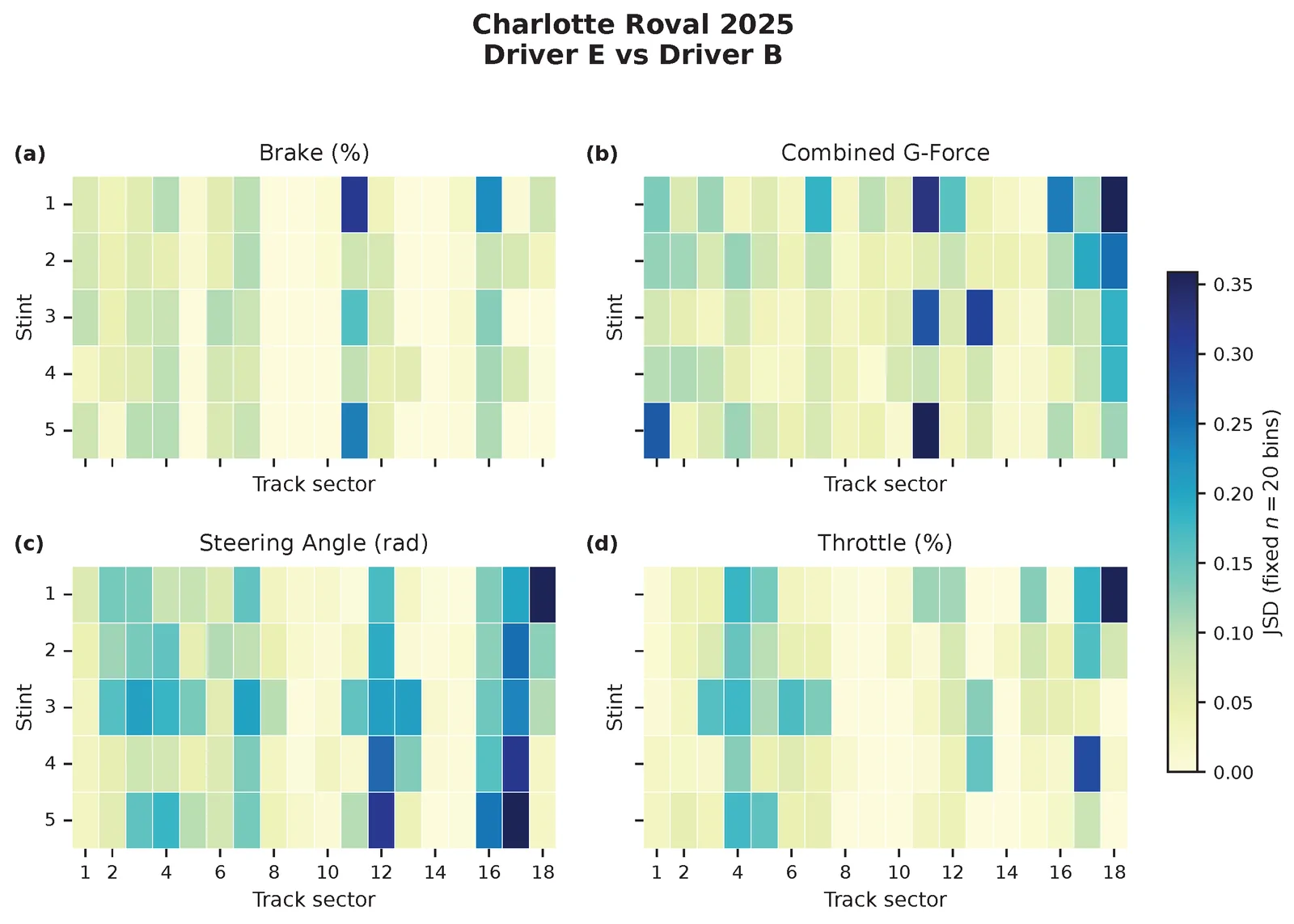
Sector × stint JSD for the uncoached driver (E) relative to Driver B at Charlotte Roval 2025.

Figure 11, Figure 12, Figure 13 and Figure 14 show the JSD map per sector (horizontal axis) and per stint (vertical axis) for the four control channels, with panels ordered (a) Brake, (b) Combined G-Force, (c) Steering Angle, and (d) Throttle.

Three structural patterns emerge. First, divergence is spatially concentrated rather than distributed: at Summit Point, Driver B vs Driver A comparison localizes steering divergence around sectors 5–8 and throttle divergence almost entirely in sector 5, while the remaining sectors stay below JSD=0.03. This confirms that the deficit is corner-specific, not a global control offset. Second, divergence magnitude scales with the skill gap: the Charlotte Roval comparisons reach JSD≈0.36, roughly twice the Summit Point ceiling, consistent with the longer and more sequenced corner combinations of the Roval layout. Third, the Combined G-Force channel diverges least across every comparison, whereas Steering Angle and Throttle carry the divergence signal.

The stint dimension separates the feedback group from the control. For the beginners (Drivers C, D, and F versus Driver B, Figure 12), the high-divergence cells in the steering and throttle panels do not persist uniformly across Stints 1–5; sector 5 at Summit Point, the dominant divergence locus in Stint 1, attenuates in later stints. Driver E, the no-feedback participant (Figure 13 and Figure 14), retains high-divergence cells in the same sectors throughout the five stints, including the strongest throttle cells at sectors 7–8 in Stint 4.

#### 6.3.2. Ordinal Complexity Relative to the Expert Reference

**Figure 15:**
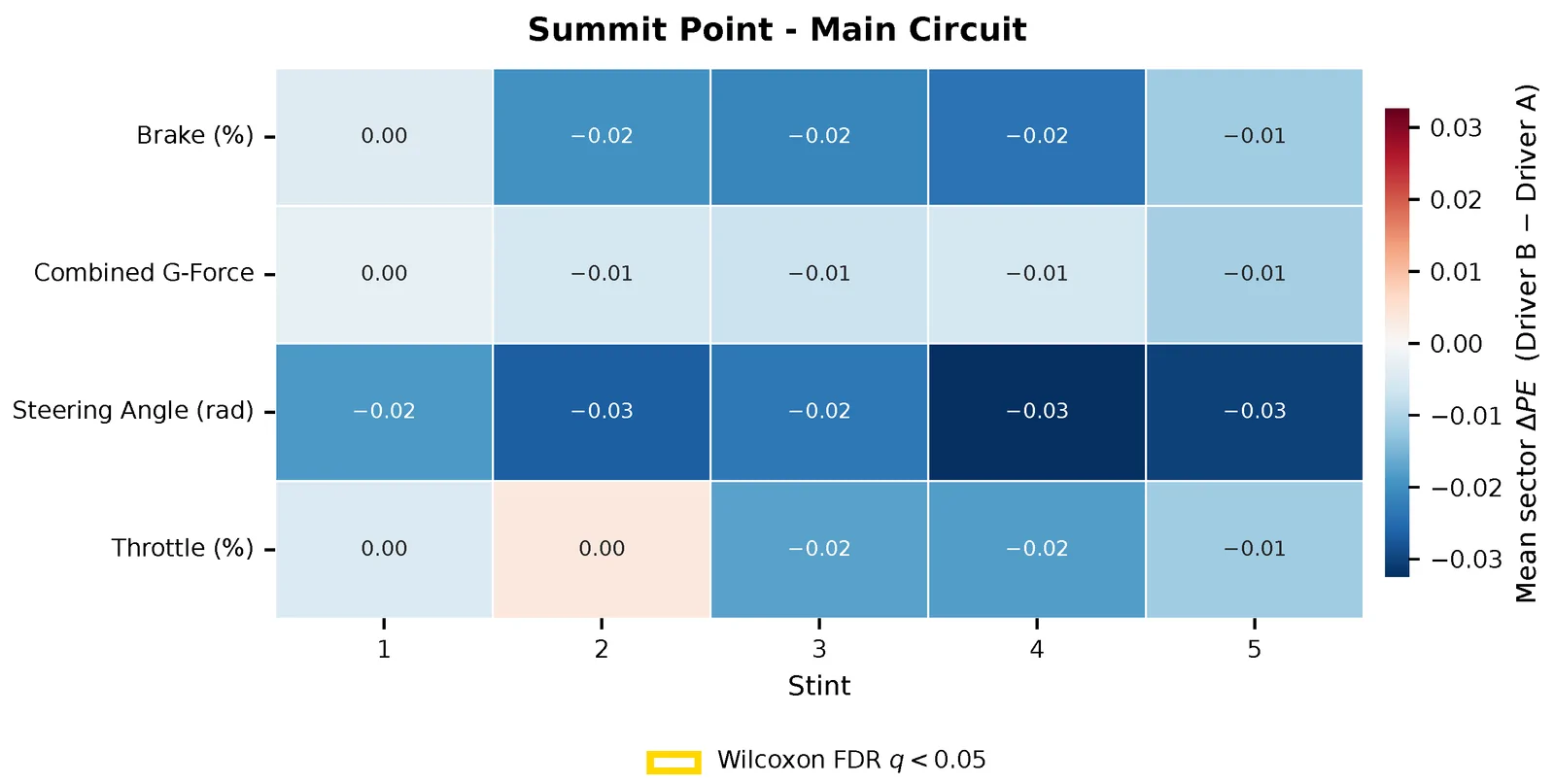
Mean sector ΔPE (Driver B − Driver A) across stints at Summit Point.

**Figure 16:**
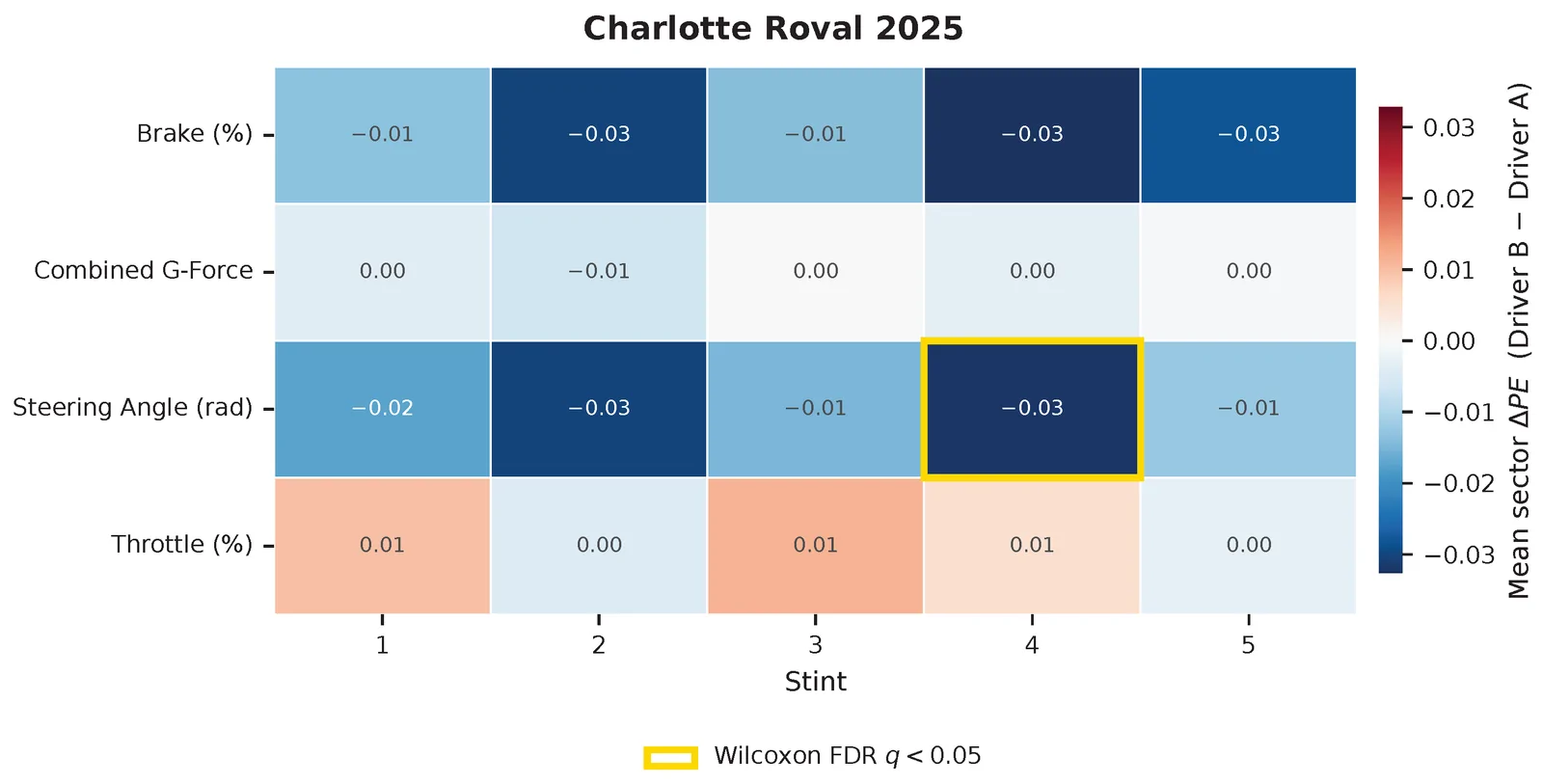
Mean sector ΔPE (Driver B − Driver A) across stints at Charlotte Roval 2025.

Figure 15 and Figure 16 report mean sector ΔPE for Driver B relative to Driver A across the five stints. Gold borders identify cells that meet the within-driver Wilcoxon FDR screening criterion (q<0.05).

The intermediate driver operates at consistently lower ordinal complexity than the expert on every channel and both tracks. At Summit Point, $\Delta PE_{\mathrm{steer}}$ ranges from −0.02 to −0.03 across all five stints, with brake and throttle following the same sign. At Charlotte Roval the steering deficit reaches −0.03 in Stints 2 and 4, the latter surviving FDR correction. This direction is informative: the expert does not drive with smoother inputs, but with a richer ordinal collection. Higher expert PE reflects continuous micro-adjustment against a moving grip limit, whereas the intermediate driver applies more repetitive, lower-variety input patterns. Combined G-Force again shows near-zero differentials (|ΔPE|≤0.01), reinforcing that the vehicle-level load outcome converges while the control strategy generating it does not.

#### 6.3.3. Heterogeneity Among Beginners and the Control Condition

**Figure 17:**
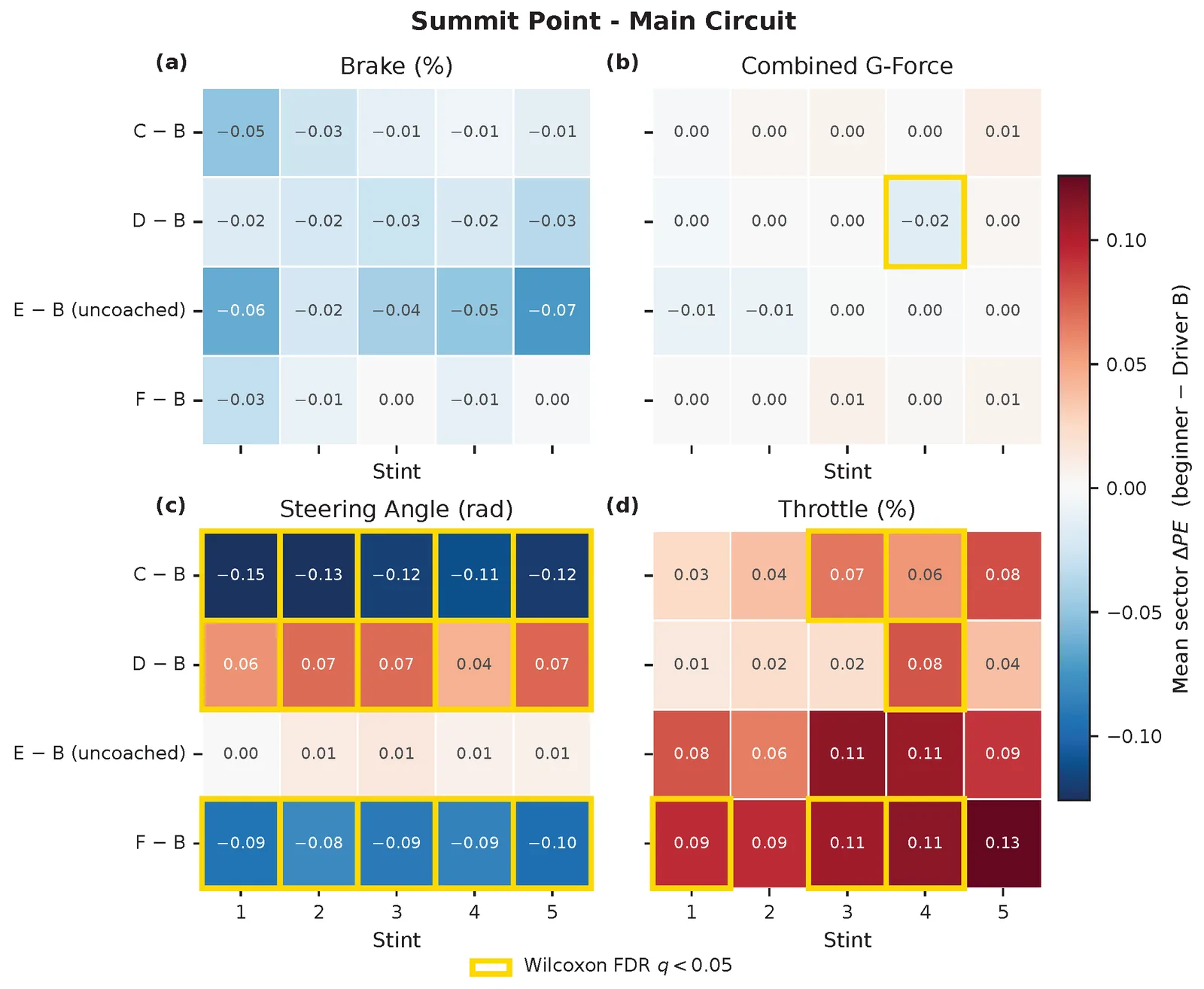
Beginner drivers’ heterogeneity across stints at Summit Point.

**Figure 18:**
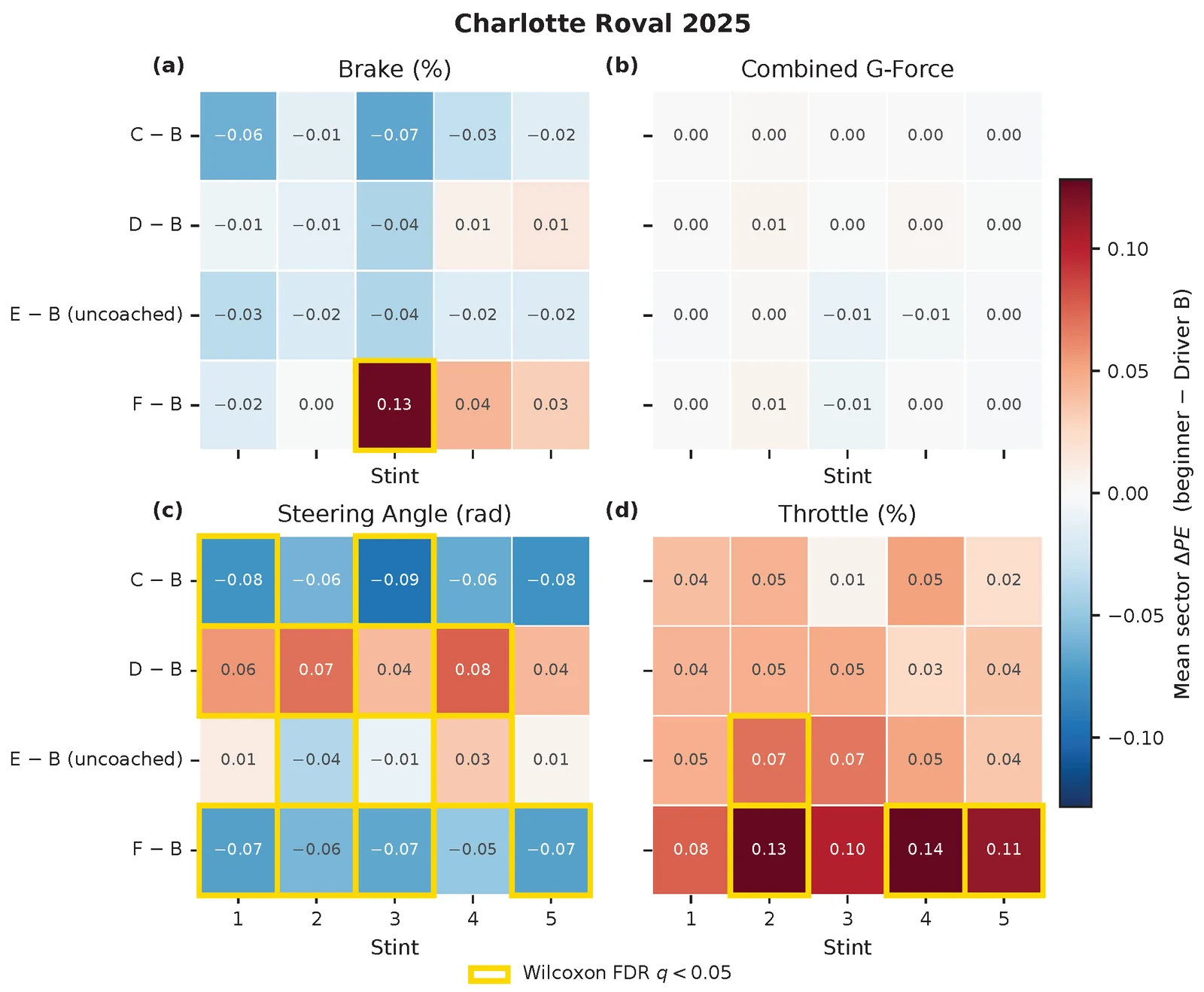
Beginner drivers’ heterogeneity across stints at Charlotte Roval 2025.

Figure 17 and Figure 18 shows the beginner group, reporting mean sector ΔPE for each driver relative to driver B, with the uncoached driver (E) shown in a dedicated row.

The beginner group is not behaviorally homogeneous. On the steering channel at Summit Point, under the within-driver FDR screening criterion, Driver C remains below the intermediate reference across all five stints, while Driver F shows the same directional pattern and Driver D remains predominantly above the reference. These repeated directional patterns are more informative than any isolated sector-wise *q*-value because the sectors are not independent experimental units. Opposite ΔPE signs on the same channel, same track, and same reference indicate two distinct failure modes: under-modulated, near-deterministic steering in Drivers C and F, versus over-corrective, high-variety steering in Driver D. A scalar skill label cannot distinguish these; the ordinal descriptor can, and the coaching prescriptions they imply are inverse.

The throttle channel, on the other hand, behaves differently. Here, every beginner sits above the reference (ΔPE>0 across both tracks), with Driver F reaching +0.13 at Summit Point in the last stint and +0.14 at Charlotte in Stint 4. This consistently positive throttle ΔPE indicates greater ordinal variability in throttle application relative to the reference. This pattern is shared across the beginner drivers in the present dataset, whereas the steering pattern remains driver-specific. Driver E, the uncoached participant, shows a similar throttle elevation (+0.06 to +0.11 at Summit Point) without the pronounced steering deviations observed for the other beginners.

**Figure 19:**
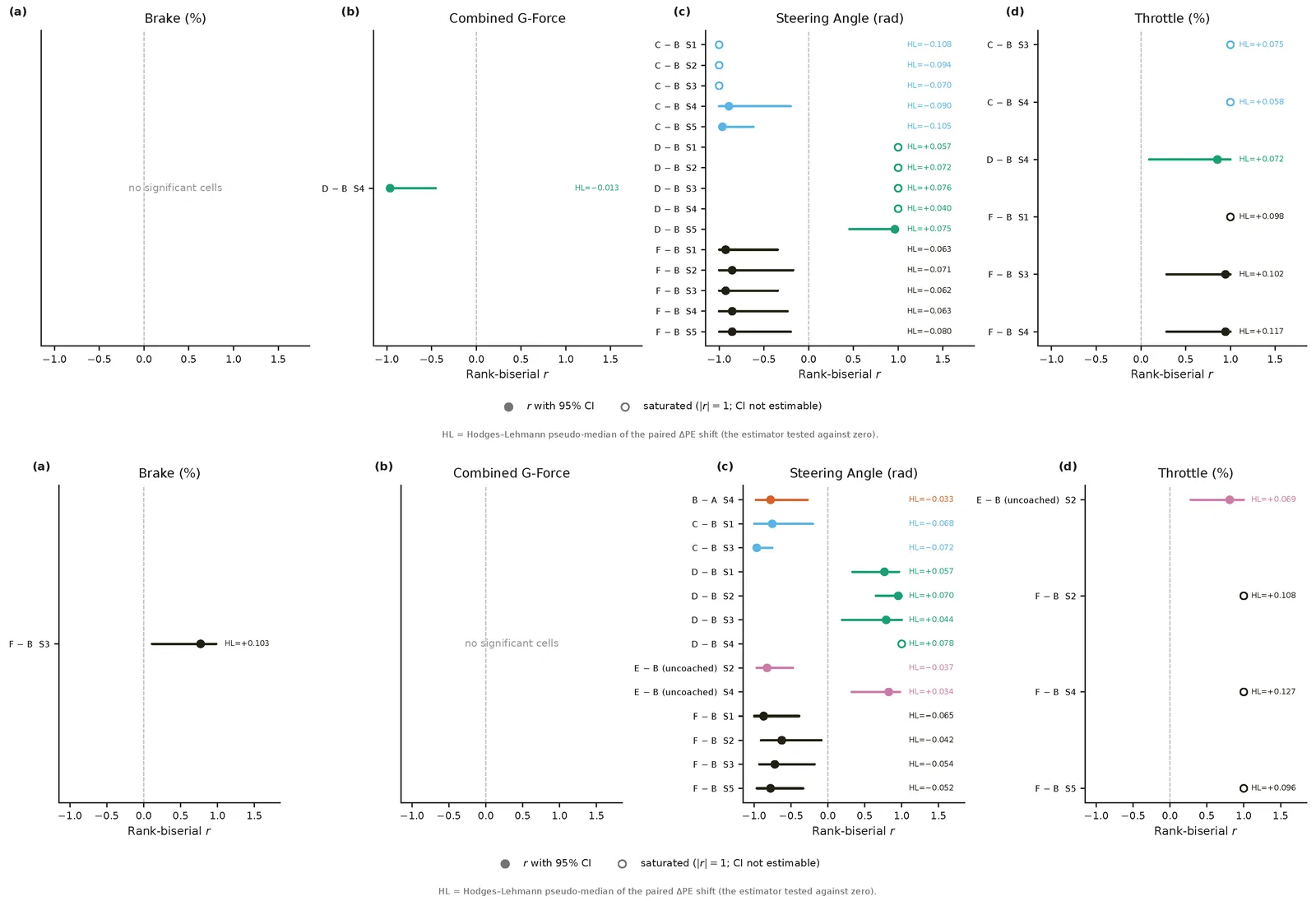
Matched -pairs rank-biserial effect sizes for significant sector-wise ΔPE cells at Summit Point (**top**) and Charlotte Roval (**bottom**), with 95% bootstrap confidence intervals (BCa, 10,000 resamples).

Figure 19 reports the effect size of every FDR-significant ΔPE cell with its 95% confidence interval—and within each observation, it is possible to see open or closed markers, where the first denotes saturated estimates (|r|=1), for which the interval is not estimable by resampling and uncertainty is read from the Hodge–Lehmann (HL) pseudo-median instead. Sectors are the matched unit within each driver; intervals quantify within-driver precision and do not support population-level inference.

Some insights can be seen. First, the steering channel concentrates nearly all significant cells on both tracks, while brake and combined G-force are almost empty—the descriptor localizes a specific control channel rather than firing indiscriminately. Then, the direction of the steering effect is a per-driver feature that replicates across circuits: Drivers C and F shift below the intermediate reference on both tracks, whereas Driver D shifts above it on both, with several cells saturated at |r|=1 (every sector moving in the same direction).

#### 6.3.4. Coupling Behavioral Distance to Lap-Time Gap

**Figure 20:**
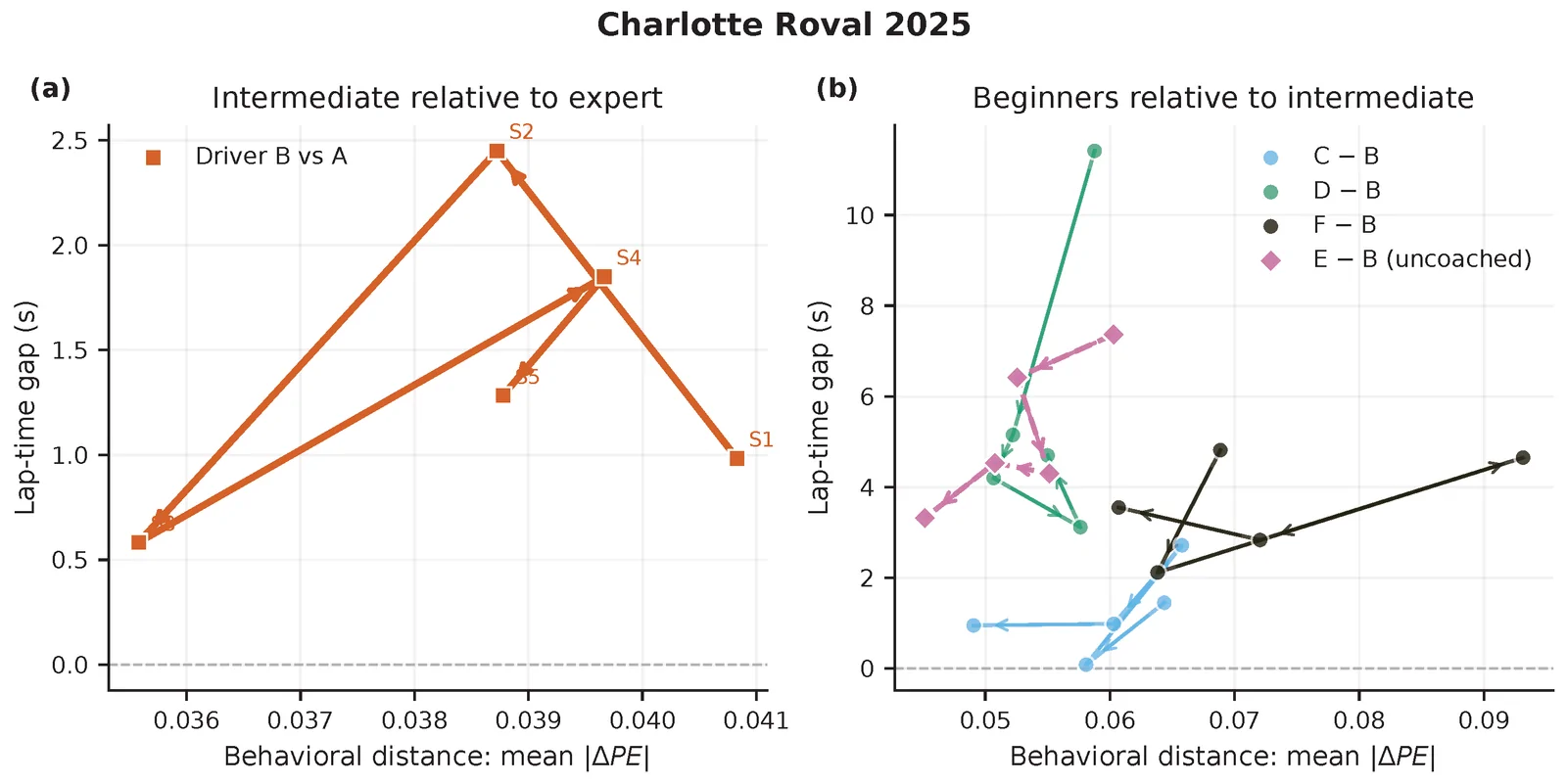
Behavioral distance (mean |ΔPE|) versus lap-time gap across Stints 1–5 at Charlotte Roval 2025: (**a**) intermediate relative to expert; (**b**) beginners relative to intermediate. Arrows indicate stint order.

Figure 20 plots each driver’s trajectory across Stints 1–5 in a two-dimensional space defined by behavioral distance (measured by the mean |ΔPE| on the X-axis) and lap-time gap in seconds (Y-axis), with arrows indicating temporal order. A trajectory moving toward the lower-left corner indicates a driver converging on the reference both behaviorally and in lap gap.

Panel (a) shows Driver B relative to Driver A at Charlotte Roval. The trajectory is non-monotonic: the lap-time gap widens to +2.45 s in Stint 2 before contracting to +0.58 s, while behavioral distance narrows from 0.041 to 0.036. The Stint 2 excursion is consistent with a transient cost of reorganizing an established control pattern—performance degrades before it recovers.

Panel (b) shows the beginners relative to Driver B. The feedback group and the uncoached driver occupy separable regions: Driver C converges to the lowest lap-time gaps (0.1–2.7 s) at moderate behavioral distance, Driver F traverses the widest behavioral range (0.060–0.093), and Driver D combines the largest lap-time gap (11.4 s in the initial stint) with a subsequent contraction to 3.1 s. The control (Driver E) moves leftward in behavioral distance while its lap-time gap oscillates between 3.3 and 7.4 s without sustained convergence. Behavioral distance and lap-time gap are therefore not interchangeable: reducing |ΔPE| does not guarantee time recovery, which is precisely why the framework reports both rather than collapsing them into a single score.

#### 6.3.5. Statistical Analysis

The density of FDR-significant cells varies substantially across drivers, and this variation is itself interpretable rather than an artifact to be smoothed over. Drivers C and F show significant steering deviations in all five stints at Summit Point, whereas Driver E, the control, shows none on that channel. Two mechanisms can produce this contrast: a genuinely smaller effect, or reduced statistical power from fewer valid laps. We separate them by inspecting the effect magnitudes directly where Driver E’s steering ΔPE never exceeds |0.01| or an order of magnitude below the −0.15 to −0.08 range observed for Drivers C and F. The absence of significance for Driver E therefore reflects a near-null effect, not an underpowered one.

The reverse case appears in the Combined G-Force panels, where all cells sit within |0.02| and only one reaches significance (Driver D, Stint 4, Summit Point). We treat this isolated cell as a candidate false positive surviving FDR rather than as a behavioral finding, since it is neither replicated across stints nor consistent with the other three channels for the same driver. Reporting it explicitly, instead of omitting it, keeps the correction procedure auditable.

We therefore interpret the sector-wise statistical tests as a screening layer rather than as confirmatory evidence. Greater weight is given to the magnitude and direction of ΔPE, persistence across consecutive stints, and recurrence of the same driver-specific pattern across the two circuits. Isolated cells meeting the q<0.05 criterion are treated as provisional when they are not supported by these additional patterns. Because the sectors are repeated and spatially ordered observations within each driver, the analyses characterize within-driver behavioral structure under the adopted sectorization and do not establish independent sector effects or population-level differences.

### 6.4. LLM Coaching Feedback and Behavioral Adaptation

As expected, the heuristic layer produces diagnostic tokens, but these tokens are not suitable for driver review. Table 9 thus presents a representative output for the Driver B versus Driver A comparison at Charlotte Roval.

In the representative example shown in Table 9, the generated text reflects the activated heuristic tokens translated into user-understandable feedback. This example illustrates the intended role of the LLM, but it does not confirm that all generated messages remain consistent with the provided diagnostic tokens nor add any new telemetry diagnosis.

After each stint run, every driver completed a short questionnaire on the Google Forms platform. The form contained three 5-point Likert items adapted from the Technology Acceptance Model [35] plus one open question for driver feedback self-evaluation. Since no validated scale exists for LLM coaching evaluation in sim racing, the items were developed by the authors and are reported here as descriptive evidence. It is important to mention that participation was voluntary and that all participants provided informed consent before the study. Additionally, no personally identifiable information was collected, and all questionnaire responses and telemetry data were anonymized prior to analysis.

**Figure 21:**
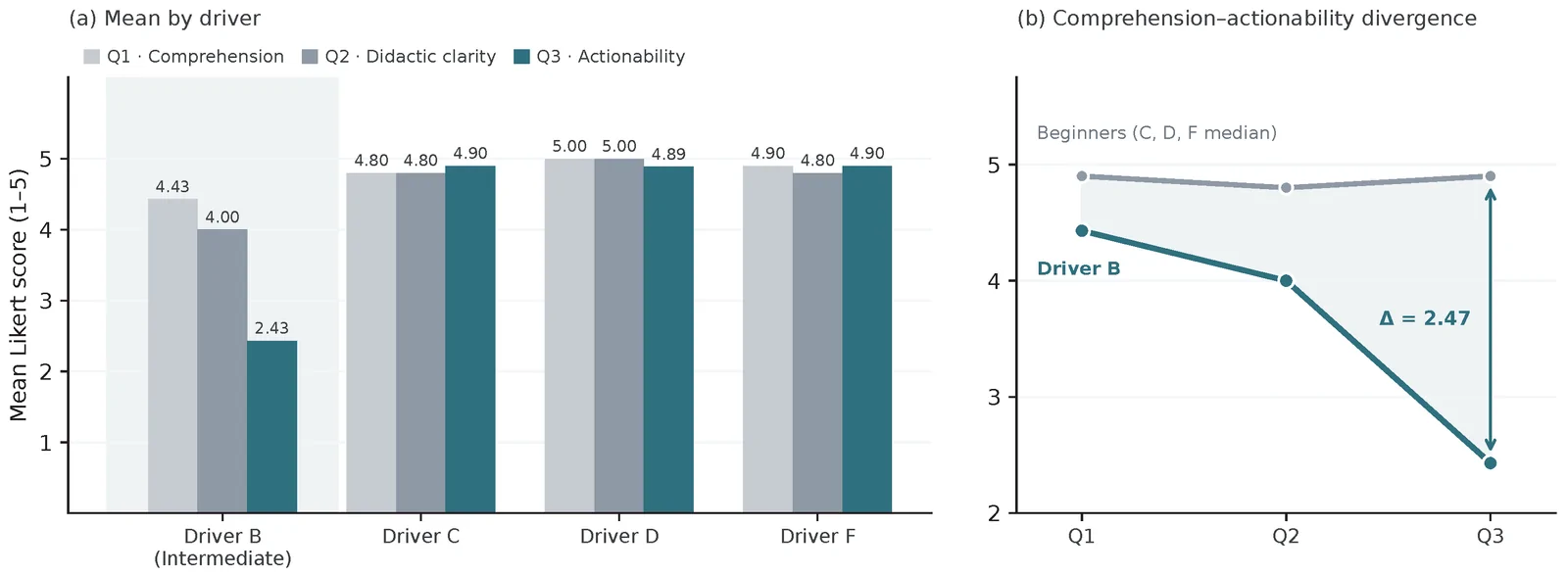
Post-feedback questionnaire results pooled across all stints and both tracks.

Figure 21 summarizes the driver feedback. The three beginners (Drivers C, D, and F) rated all items near the scale ceiling (means between 4.80 and 5.00). Driver B rated comprehension and clarity highly (Question 1 =4.43; Question 2 =4.00) but actionability poorly (Question 3 =2.43), a gap of 2.5 points relative to the beginner median. Within his open-ended answers, it was said that recommendations perceived as imperceptible at the control level (e.g., steering corrections of 5°), the absence of quantitative targets such as brake-pressure values or braking distances in meters, and one contradictory braking recommendation issued after the suggested correction had already been implemented. Thus, we expect that an intermediate driver operating near the vehicle limit may require more precise numerical feedback than natural-language recommendations alone [15,25].

These subjective results corroborate the expected limitation that was observed previously, which is the fact that an intermediate driver may not properly convert the received feedback into measurable control changes, an interesting initial conclusion that might guide further research in this area.

The evaluation of the semantic layer relies on subjective driver ratings and therefore does not quantify the fidelity of the generated text with respect to the diagnostic tokens that produced it. A stricter assessment, comparing the generated recommendations against coaching reports written manually for the same sessions, or rating each statement for technical correctness, requires a qualified driving coach to independently annotate every message, sector by sector and stint by stint, across all six drivers and both circuits. This annotation effort falls outside the scope of the present feasibility study. We therefore report the semantic layer as functional rather than validated and restrict our claims.

### 6.5. Post-Feedback Performance Adaptation

**Figure 22:**
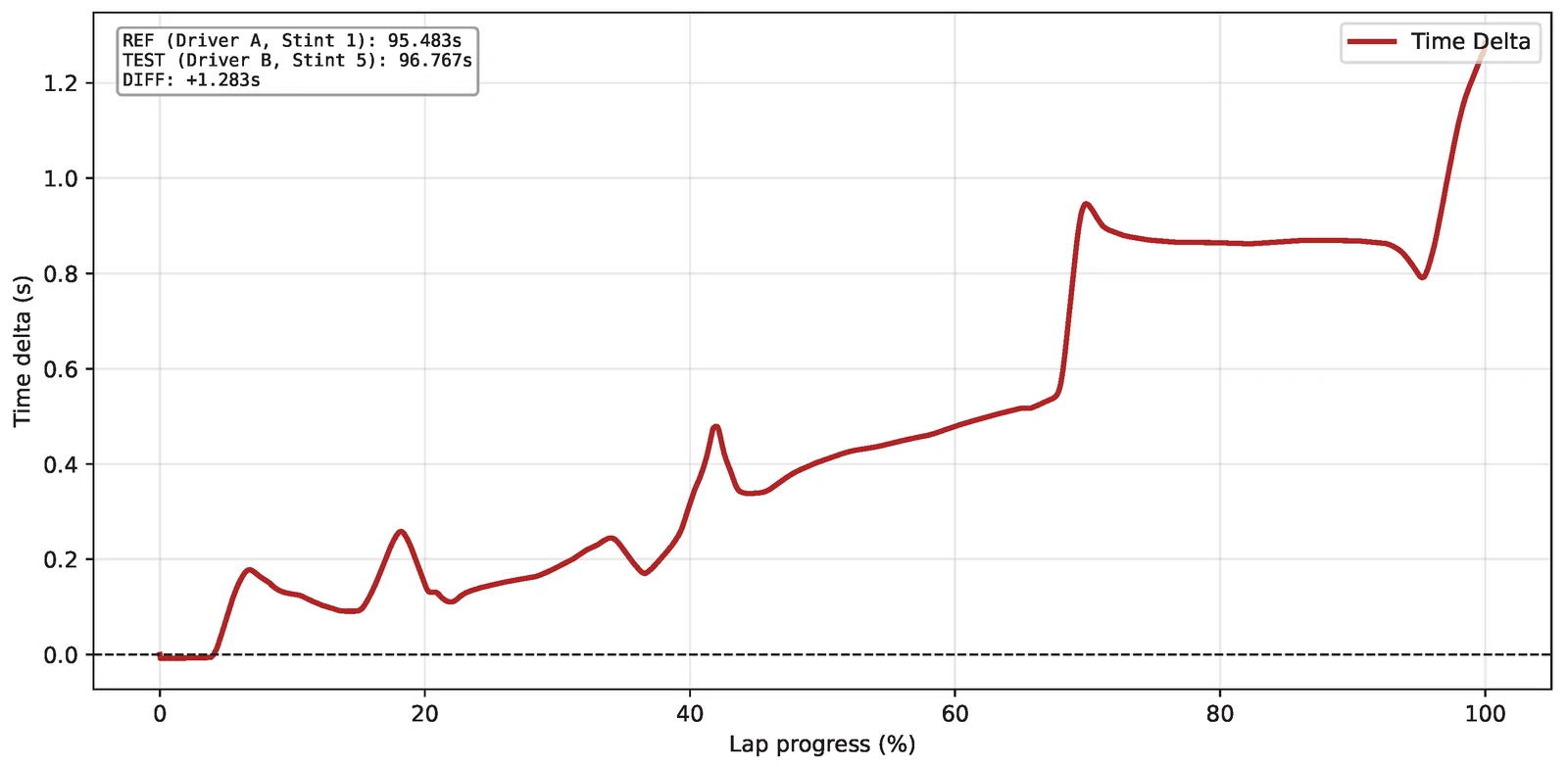
Continuoustime-delta profile for Driver B vs. Driver A for Stint 5 at Charlotte Roval.

**Figure 23:**
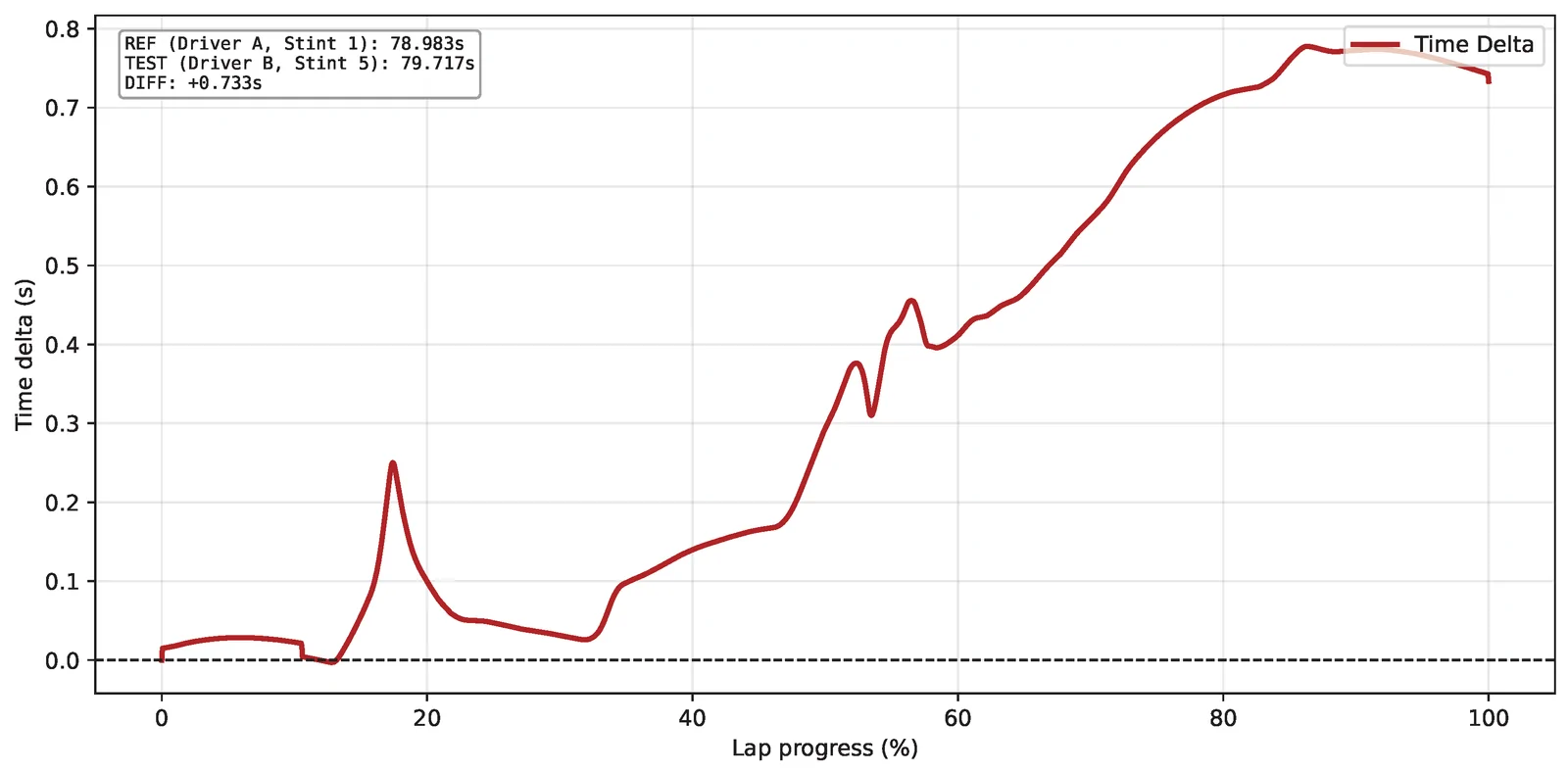
Time-delta profile for Driver B vs. Driver A for Stint 5 at Summit Point.

**Figure 24:**
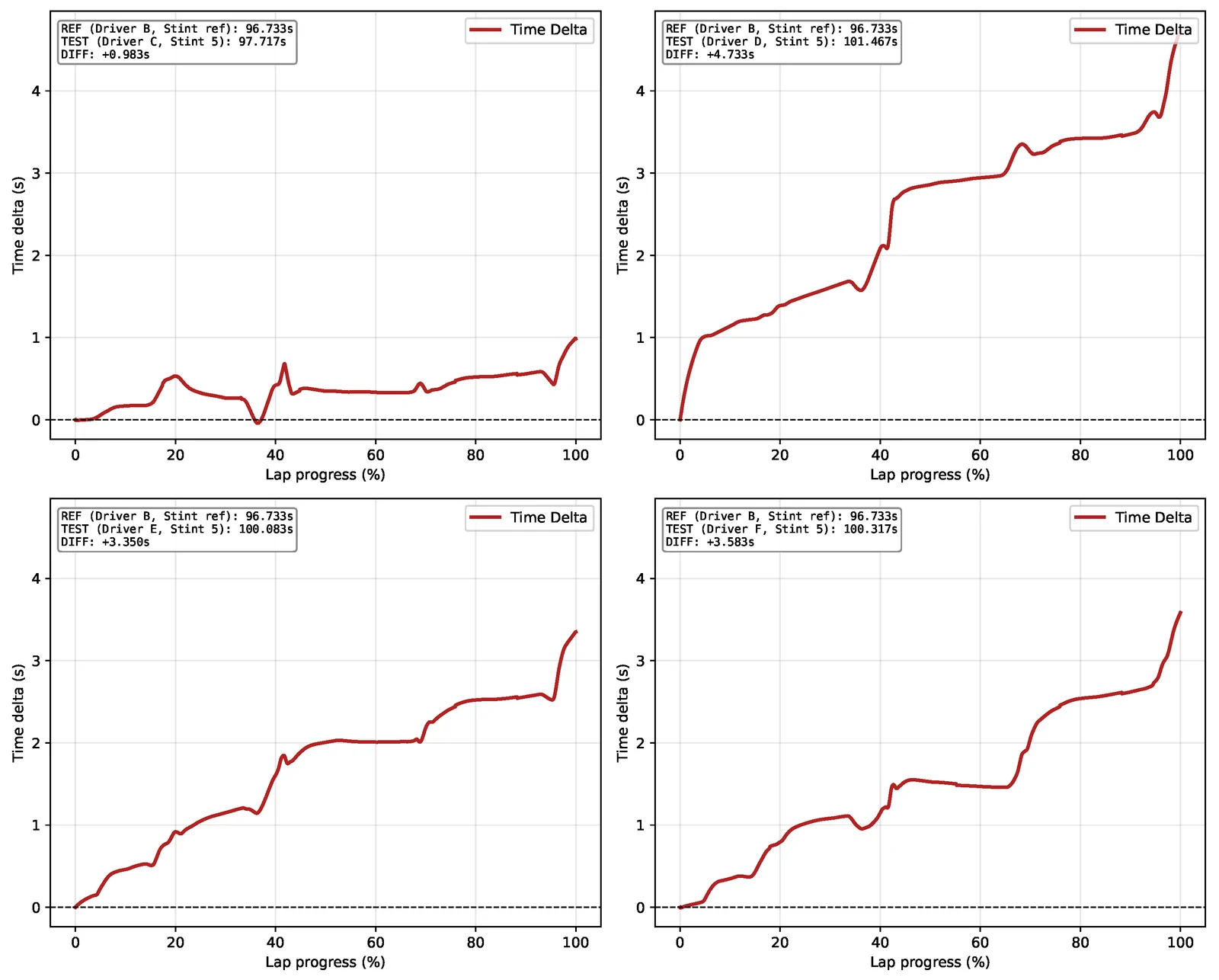
Time-delta profiles for beginner drivers (C, D, E, F) vs. the intermediate reference (Driver B) for Stint 5 at Charlotte Roval.

**Figure 25:**
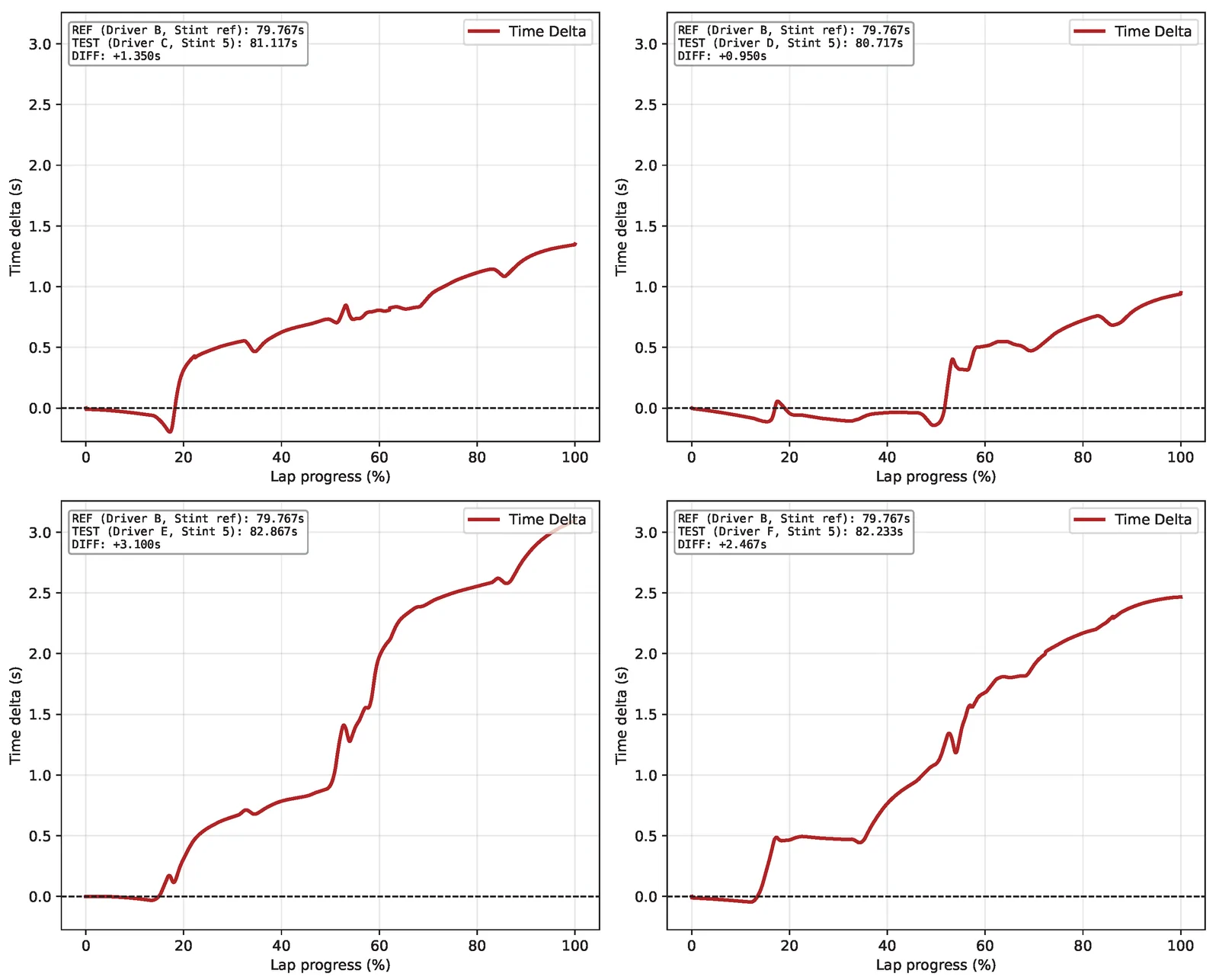
Time-delta profiles for beginner drivers (C, D, E, F) vs. the intermediate reference (Driver B) for Stint 5 at Summit Point.

The performed comparisons tell only part of the story. To assess whether the observed change persists, we tracked the lap-time gap to the intermediate reference (Driver B) across all five stints for the four beginner drivers, three of whom received LLM-generated feedback (Drivers C, D, and F) and one of whom did not (Driver E). Figure 22, Figure 23, Figure 24 and Figure 25 present these additional results.

Table 10 reports the initial (Stint 1) and final (Stint 5) gaps. At Charlotte Roval, the three coached beginners converged toward the reference: Driver C reduced the gap from +1.183 s to +0.983 s (−16.9%), Driver D from +11.150 s to +4.733 s (−57.6%), and Driver F from +4.550 s to +3.583 s (−21.3%). Nevertheless, the uncoached participant also reduced the lap-time gap, from +7.100 s to +3.350 s (−52.8%). This trajectory shows that substantial lap-time changes occurred without generated coaching messages, but the design does not identify whether the change resulted from practice, familiarization, run-to-run variation, or participant-specific adaptation.

Analyzing the Summit Point track, we can see that it separates the two groups more clearly, where Driver C reduced the gap from +2.333 s to +1.350 s (−42.1%) and Driver D from +2.817 s to +0.950 s (−66.3%), whereas Driver F decreased his time gap from +2.867 s to +2.467 s only marginally (−14.0%). Unlike the Charlotte track, the uncoached driver diverged here, while he had a time-gap reduction of −3.750 s at Charlotte; at Summit Point, his gap was not significant compared to his first stint (−0.467 s).

The Intermediate–Expert pair follows the opposite direction on both circuits. While Driver B’s gap to Driver A widened from +0.983 s to +1.283 s at Charlotte Roval, at Summit Point it varies from +1.600 s to +0.733 s. This inconsistency suggests the proficiency-dependent limitation is already visible in the questionnaire responses (Section 6.4): qualitative recommendations perturb an already-consolidated control strategy without supplying the numerical references needed to replace it.

**Figure 26:**
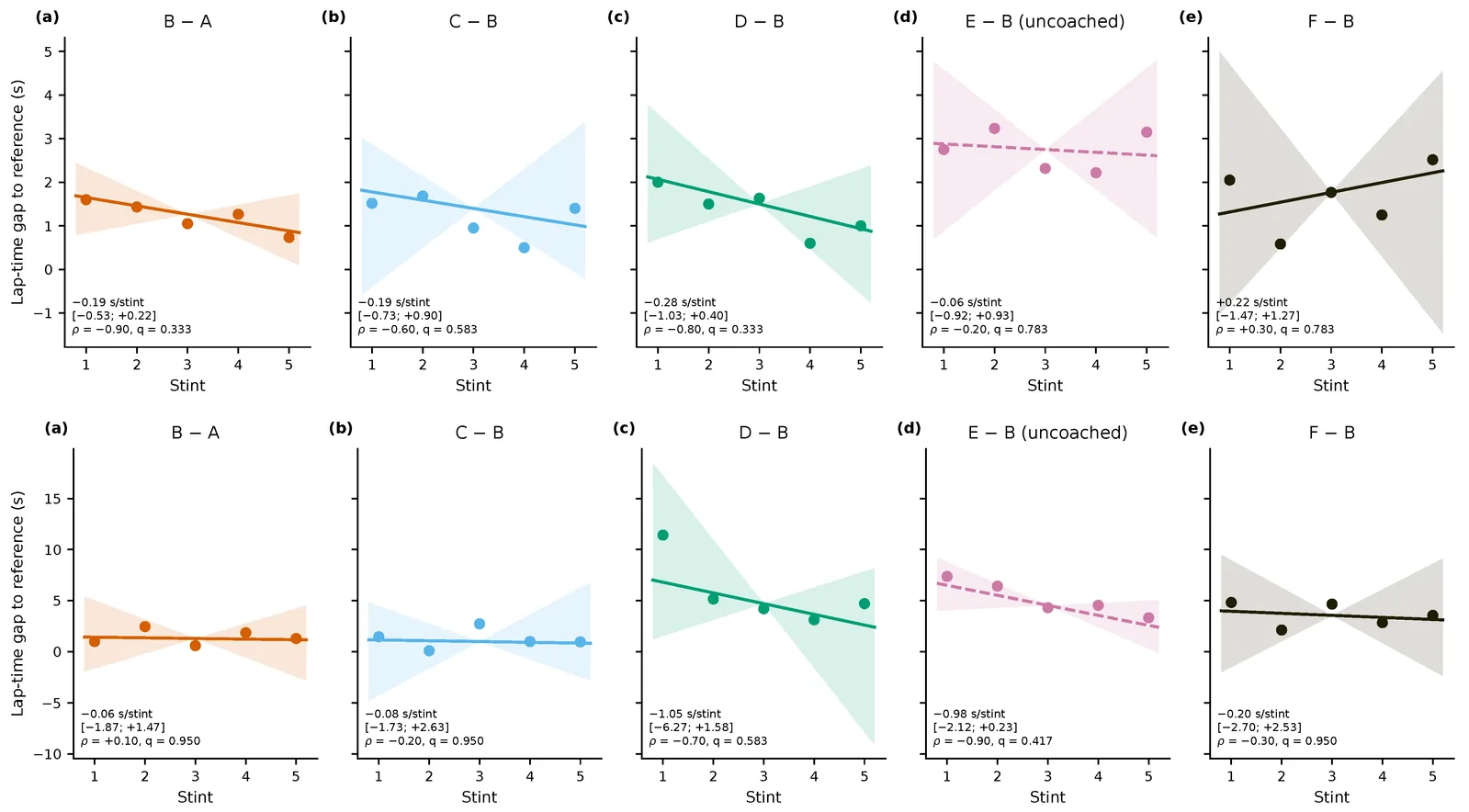
Lap-time gap to the proficiency-matched reference across stints at Summit Point (**top**) and Charlotte Roval (**bottom**).

Moreover, we tested each driver’s lap-time trajectory with an exact permutation test on Spearman’s ρ across the five stints, paired with Theil–Sen slope estimates shown in Figure 26. No trend reaches significance after FDR correction, and every slope interval crosses zero. This null result is related to the smallest attainable *p* (2/120≈0.017) within the five stints, which after FDR correction across all drivers cannot fall below q≈0.083—a perfectly monotonic improvement by a single driver is insufficient by design. We therefore report the per-driver slopes with their intervals as descriptive evidence and treat lap time strictly as a secondary outcome.

**Figure 27:**
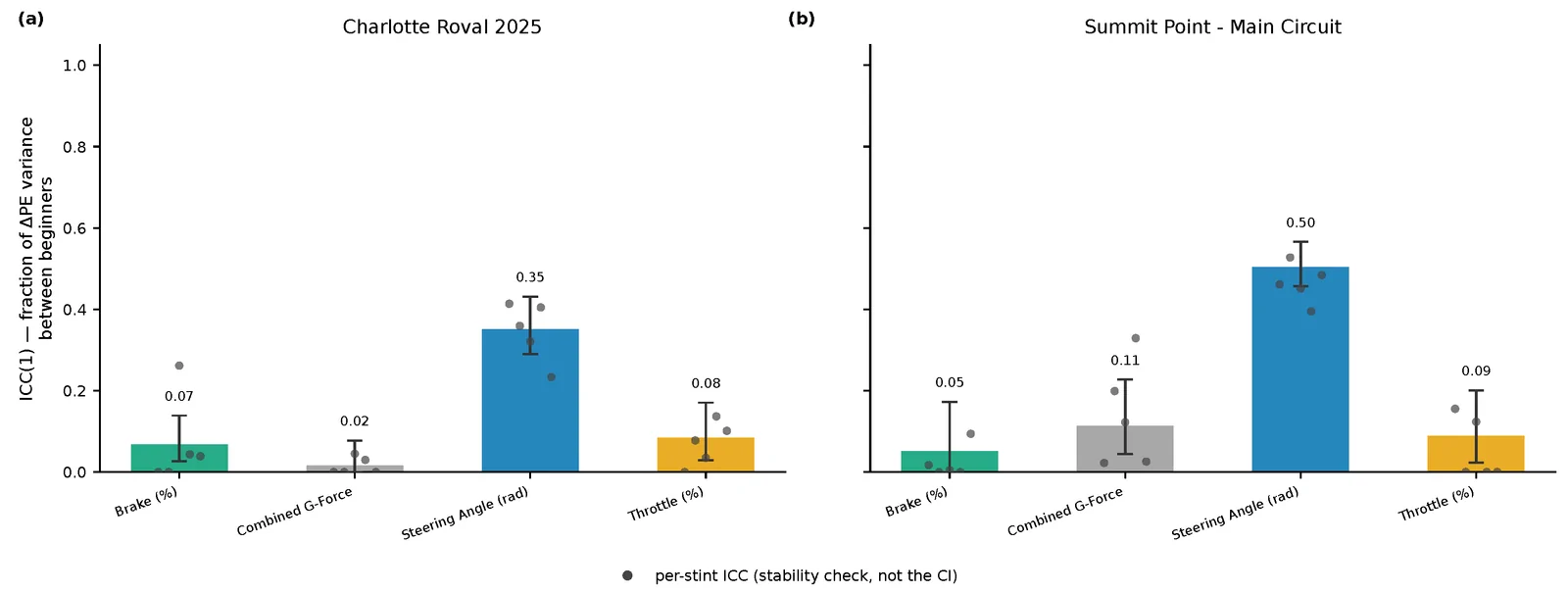
Inter-driver variability of sector-level ΔPE among beginners. Bars show the median ICC(1) across stints per channel and track.

The heterogeneity visible in Figure 17 and Figure 18 can be quantified. Figure 27 shows a one-way random-effects decomposition of sector-level ΔPE, expressed as the intraclass correlation ICC(1)—the fraction of variance attributable to differences between beginners. Steering carries the highest and most stable ICC on both tracks (0.36 at Charlotte Roval, and 0.46 at Summit Point, with narrow min–max ranges across stints), against values near zero for throttle and brake. The two extremes carry complementary meanings. Steering is the individual channel: it is where coached beginners diverge most from one another, including in direction—Drivers C and F reduce steering ordinal complexity toward the reference while Driver D consistently increases it—so the coached-group median understates every member simultaneously and must not be read as a typical response. Combined G-force is the collective channel: an ICC near zero indicates that between-driver variance was small relative to within-driver sector variance in this dataset; it does not establish equivalence among the beginner drivers.

### 6.6. Post-Feedback Stochastic Evolution

The lap-time convergence reported in Section 6.5 cannot, by itself, distinguish whether drivers may have restructured their control behavior (due to our solution), or simply drove faster through practice. The stochastic descriptors introduced in Section 6.3.1, Section 6.3.2 and Section 6.3.3 provide complementary operational indicators of whether the statistical organization of the telemetry signals changed across stints. However, these descriptors characterize the observed change and do not identify whether it was caused by coaching, practice or participant-specific adaptation.

At the distribution level, contraction is observed in the coached beginner trajectories. For them, as depicted in Figure 12, the dominant divergence found in Stint 1, mostly in the throttle cell at sector 5 of Summit Point, attenuates across Stints 2–5, alongside the lap-time gains reported in Table 10. Driver E also improved lap time at Charlotte Roval but retained several high-divergence regions across stints (Figure 13 and Figure 14). This contrast is consistent with the hypothesis that coaching may be associated with behavioral reorganization beyond practice-related performance gains. However, because the uncoached condition contains one non-randomized participant, the observed contrast cannot establish a causal effect of coaching.

Moreover, ΔPE provides a quantitative within-driver view of this contrast rather than relying only on visual inspection. The gold-bordered cells in Figure 17 and Figure 18 (Wilcoxon signed-rank, Benjamini–Hochberg q<0.05) concentrate on the steering channel of the coached beginners: Driver C remains significantly below the intermediate reference in all five stints at Summit Point, with the deficit contracting from −0.15 to −0.12, while Driver D’s over-corrective surplus contracts from +0.07 to +0.04 before rebounding. Driver E’s steering cells never reach significance at Summit Point, and at Charlotte Roval, his two significant steering cells carry opposite signs (Stint 2: −0.037; Stint 4: +0.034). In Figure 19, we see uncoached driver steering input oscillating without direction, in contrast with the coached drivers, whose significant cells maintain a consistent sign across stints and across both circuits.

The throttle channel shows a different response profile. All four beginners, coached and uncoached alike, remain above the reference in ordinal complexity through Stint 5 (ΔPE>0 in every cell of panels (d) in Figure 17 and Figure 18), and Driver F’s elevation grows to +0.13 at Summit Point. Hesitant, repeatedly modulated throttle application persists as the shared beginner signature that five stints of qualitative feedback did not erase—consistent with traction management being the last skill consolidated in the proficiency hierarchy.

The intermediate driver completes the picture. Driver B’s behavioral distance to the expert narrows across stints (Figure 20a, mean |ΔPE| from 0.041 to 0.036) even as his Charlotte Roval lap-time gap widens from +0.983 s to +1.283 s (Table 10), and only a single cell—steering at Stint 4, Charlotte Roval (Figure 16)—survives FDR correction. Behavioral convergence without time recovery inverts Driver’s E pattern and corroborates the questionnaire evidence of Section 6.4: Driver B assimilates the recommendations at the ordinal-structure level but cannot convert them into lap time near the vehicle limit. Table 11 consolidates the stochastic and performance evidence discussed in this section into a single per-driver synthesis.

Overall, the post-feedback stochastic evolution reveals three complementary patterns that cannot be captured by lap time alone: changes in the underlying probability distributions (JSD), changes in the ordinal organization of driving inputs (ΔPE), and changes in lap time. For the ΔPE results, interpretation emphasizes effect magnitude, directional consistency, persistence across stints, and recurrence across circuits, while sector-wise FDR results are used as complementary within-driver screening information. Finally, given the small sample size (N=6 drivers), these findings should be interpreted as evidence of within-subject behavioral evolution rather than population-level effects.

## 7. Conclusions

This article presented a Behavior-First Telemetry framework for the interpretable analysis of driver behavior in sim racing. The framework couples telemetry extraction, stochastic characterization, a physics-informed diagnostic layer, and an LLM feedback layer that translates deterministic tokens into coaching language without performing telemetry inference. Besides recognizing the potential of LLMs for problem solving in many scenarios [36,37], this paper advanced knowledge in this field by combining post-session telemetry and user-understandable LLM feedback, potentially supporting driving support and training.

We evaluated the proposed framework with six drivers across two circuits and five consecutive stints, spanning expert, intermediate, and amateur proficiency. The beginner group included one participant who received no coaching feedback throughout the protocol, serving as an uncoached comparison condition. Two information-theoretic descriptors carried out the analysis: Jensen–Shannon divergence, which quantifies distributional distance between a driver and a proficiency-matched reference, and permutation entropy (m=3, τ=1), which measures ordinal complexity of the control signal. Sector-level ΔPE shifts were additionally examined using paired Wilcoxon signed-rank tests with Benjamini–Hochberg FDR control (q<0.05).

The exploratory comparison revealed distinct combinations of performance and behavioral evolution. The coached beginners generally showed lap-time improvement accompanied by changes in JSD or permutation-entropy descriptors, whereas the single uncoached participant improved lap time at Charlotte Roval (from +7.100 s to +3.350 s) without comparable changes in some behavioral descriptors. These observations show that lap-time change and telemetry-structure change are not interchangeable outcomes. However, no statement in this article should be read as an estimate of the effect of the framework on driving performance. The design supports a single claim: the proposed descriptors are sensitive enough to register session-to-session behavioral change, which is a precondition for, but not evidence of, coaching effectiveness.

Two limits of qualitative coaching emerged. Throttle ordinal complexity remained above the reference for all four beginners through Stint 5, identifying traction management as the skill that verbal feedback did not consolidate. Moreover, the intermediate driver’s mean |ΔPE| moved towards the expert reference (from 0.041 to 0.036), a descriptive shift that indicates direction only, while his lap-time gap at Charlotte Roval widened, and he rated the recommendations as comprehensible but not actionable. This combination is consistent with qualitative recommendations being insufficient to replace an already-consolidated control strategy near the friction limit, which would require explicit numerical references.

An exploratory stationarity diagnostic complemented these descriptors by applying the joint ADF/KPSS procedure directly to the original 60 Hz telemetry sequences within each lap and analytical sector. The diagnostic further showed that the intra-sector telemetry is predominantly characterized by Unit-Root-compatible outcomes across drivers and channels, while Stationary-compatible outcomes represent a smaller fraction of the classifiable segments. Stationarity verdicts were not used as inclusion criteria for the PE or JSD analyses.

Some constraints bound our findings. With N=6 drivers, the study characterizes within-subject behavioral evolution rather than population-level effects; with a single non-randomized uncoached participant, the between-group variance is not estimable and no standardized treatment-versus-control contrast can be computed. Per-driver lap-time trends are instead reported individually with exact permutation tests, whose attainable significance is bounded by the five-stint design (q≥0.083 after FDR correction), which is why we treat lap time as a secondary outcome and the entropy descriptors as the primary one; the heuristic thresholds are expert-elicited and vehicle- and circuit-specific, so the diagnostic layer remains exploratory until a sensitivity analysis establishes its transferability.

Future work will extend the framework to larger driver group and additional circuits, as well as report a threshold sensitivity analysis for the heuristic layer. In addition, the heuristic rules will be independently validated against sector-level judgments provided by professional driving coaches and evaluated across additional vehicle-track configurations. Future data collection with longer within-session sequences will also allow stability of the intra-sector ADF/KPSS verdicts to be assessed across repeated sector runs. This expanded data will enable statistical models to track changes and compare performance across laps, sectors, channels, and drivers, while considering how neighboring track regions affect each other. The present exploratory results therefore provide a baseline for defining the data requirements of this future validation stage.

Furthermore, we also want to a migrate the post-session pipeline to a streaming architecture capable of sub-second intervention. The proficiency-dependent limitation pointed out by an intermediate driver motivates a quantitative feedback mode for experienced drivers, in which heuristic tokens carry explicit numerical targets rather than qualitative descriptors. Moreover, we will also investigate the integration of advanced LLM agent frameworks, such as ReAct, Reflexion, and reflective multi-agent systems, to evaluate how autonomous reasoning and self-reflection mechanisms can enhance decision-making beyond the semantic translation capabilities explored in this study.

A dedicated evaluation of the semantic layer is also planned. In this follow-up, a professional driving coach will independently write reference reports for the same sessions and rate each generated statement for technical fidelity, completeness, and actionability, allowing the generated text to be scored against both a human baseline and a deterministic rule-based one. Moreover, the same study will quantify the run-to-run stability of the layer, submitting each payload to *n* independent generations under fixed decoding parameters, and agreement will be measured over the diagnostic content asserted in each report.

Finally, future studies will investigate multimodal entropy across concurrent control channels and evaluate the framework on real-world motorsport telemetry, potentially enlarging the contributions to the field. 

## Figures and Tables

**Table 1:** Methodological and analytical comparison of related works.

Study	Primary Objective	Analytical Approach	Feedback and LLM Role	Platform
[9]	Real-time training	Rule-based telemetry analysis	Auditory feedback, no LLM	Custom simulator
[5]	Behavior benchmarking	Clustering	No feedback, no LLM	Professional simulator
[6]	Skill classification	Machine learning classification	No feedback, no LLM	ACC
[7]	Performance modeling	Clustering and telemetry modeling	No feedback, no LLM	ACC
[24]	Autonomous driving	Deep RL (PPO) and vehicle states	No feedback, no LLM	TORCS
[14]	Literature review	Review of driver behavior classification	Not applicable	Not applicable
[15]	Multimodal coaching	Experimental What/Why explanations	AI-assisted visual and auditory feedback	Research simulator
[16]	Driver coaching	Counterfactuals and SHAP	LLM-based coaching generation	IPG CarMaker
[25]	Interactive coaching	Numerical gap comparisons	Conversational AI interface	CARLA
This work	Behavior-aware coaching	Stationarity, entropy, and heuristics	Constrained semantic interface	iRacing

**Table 2:** Per-sector kinematic descriptors extracted from the aligned telemetry channels. Symbols match the trigger conditions of Table 3.

Phase	Descriptor	Description	Formula	Unit
Sector Boundary	$V_{\mathrm{start}}$	Instantaneous speed at the first sample of the sector (sector-entry speed).	$v_{1}$	km/h
	$V_{\mathrm{end}}$	Instantaneous speed at the last sample of the sector (sector-exit speed).	$v_{n}$	km/h
	$V_{\min}$	Minimum speed reached within the sector, typically near the corner apex.	$\min_{i}v_{i}$	km/h
	$\Delta V_{\mathrm{start}}$	Entry-speed deficit inherited from the previous sector, used by the legacy rule.	$V_{\mathrm{start}}^{U}-V_{\mathrm{start}}^{R}$	km/h
Braking	$P_{\mathrm{brake}}$	Peak brake-pedal input recorded in the sector.	$\max_{i}b_{i}$	%
	${\mathrm{Dist}}_{\mathrm{start}}$	Lap-distance fraction at which sustained braking begins: the first point where brake input exceeds 5% for at least five consecutive samples.	$\min\{\;s_{i}:b_{j}>5\%\;\forall j\in \left[i,i+5\right)\;\}$	–
	${\mathrm{Rate}}_{\mathrm{ramp}}$	Aggressiveness of the initial brake application: the 95th percentile of the brake-rate distribution while braking.	$Q_{0.95}\;\left({\dot{b}}_{i}\right),\;i\in M_{b}$	%/s
	${\mathrm{Cons}}_{\mathrm{brake}}$	Consistency of brake-rate application; higher values indicate more erratic modulation.	$\mathrm{Var}\;\left({\dot{b}}_{i}\right),\;i\in M_{b}$	(%/s)^2^
	${\mathrm{Eff}}_{\mathrm{brake}}$	Deceleration achieved per unit of brake pressure applied; low values suggest wheel lock or ABS overuse.	$\left|\min_{i\in M_{b}}a_{i}\right|/\max_{i\in M_{b}}b_{i}$	m s^−2^/%
Rotation	$\Delta_{\mathrm{MRP}\text{-}\mathrm{Vmin}}$	Timing offset between maximum rotation and minimum speed; negative values indicate the car rotates after the apex.	$t_{\arg\;\min_{i}v_{i}}-t_{\arg\;\max_{i}\left|\omega_{i}\right|}$	s
	Trail	Cumulative overlap between brake and steering inputs, quantifying how much braking is carried into the turn.	$\sum_{i}b_{i}\;\left|\delta_{i}\right|\;\Delta t$	%·rad·s
	RotEff	Yaw rotation produced per unit of steering input; low values indicate understeer.	$\frac{mean(|\omega_{i}|)}{mean|\delta_{i}\left|\right)}$	s^−1^
Steering/Traction	${\mathrm{RMS}}_{\mathrm{steer}}$	Overall steering activity in the sector; high values indicate corrections or sawing at the wheel.	$\sqrt{\frac{1}{n}\sum_{i}\delta_{i}^{2}}$	rad
	Smooth	Smoothness of throttle application; low values indicate abrupt or uneven throttle inputs.	$1/\mathrm{Var}\;\left({\dot{\tau }}_{i}\right),\;i\in M_{\tau }$	(s/%)^2^
	Conflict	Simultaneous demand for throttle and steering, indicating the driver requests traction and rotation at once.	$mean(\tau_{i}\;|\delta_{i}|)$	%·rad

**Table 3:** Summary of core physics-informed heuristics, where *U* denotes the user lap and *R* the reference lap. All thresholds are expert-elicited (Section 4.2) and follow the unit convention stated below.

Category	Heuristic ID	Trigger Condition	Physical Interpretation
Braking	BRAKING_POINT_MISMATCH	$|{\mathrm{Dist}}_{start}^{U}-{\mathrm{Dist}}_{start}^{R}|>$ 0.5% of lap distance	Severe early or late braking relative to the reference sector.
	OVER_BRAKING_PEAK	$P_{brake}^{U}>P_{brake}^{R}+15\;p.p.$	Excessive brake pressure, which may cause over-slowing or instability.
	LOW_BRAKE_EFFICIENCY	${\mathrm{Eff}}_{brake}^{U}<0.85\times {\mathrm{Eff}}_{brake}^{R}$	High brake pressure with low deceleration, suggesting locking or ABS overuse.
	AGGRESSIVE_UNSTABLE_BRAKE	${\mathrm{Rate}}_{ramp}^{U}>1.25\times {\mathrm{Rate}}_{ramp}^{R}\;\wedge \;{\mathrm{Cons}}_{brake}^{U}>1.30\times {\mathrm{Cons}}_{brake}^{R}$	Abrupt and inconsistent initial brake application.
	ENTRY_OVER_SLOW	$\frac{V_{min}^{U}-V_{min}^{R}}{V_{min}^{R}}<-0.015\;\wedge \;P_{brake}^{U}>5\%$	Driver below reference speed despite meaningful brake pressure.
Rotation	LATE_ROTATION	$\Delta {\mathrm{MRP}}_{V_{min}}^{U}<-0.05\;s$	Maximum rotation occurs after the minimum-speed point.
	POOR_TRAIL_BRAKING	${\mathrm{Trail}}^{U}<0.70\times {\mathrm{Trail}}^{R}\;\wedge \;V_{min}^{U}<V_{min}^{R}$	Early brake release before rotation, reducing front-end grip.
	STEER_EFFICIENCY	${\mathrm{RMS}}_{steer}^{U}>1.20\times {\mathrm{RMS}}_{steer}^{R}$	Excessive steering activity, causing tire scrub and speed loss.
	ROTATION_INEFFICIENT	$\Delta {\mathrm{RotEff}}^{U-R}<-0.05\;s^{-1}\;\wedge \;V_{min}^{U}<0.97\times V_{min}^{R}$	Excessive steering input fails to produce proportional rotation, indicating understeer.
Traction	THROTTLE_STEER_CONFLICT	${\mathrm{Conflict}}^{U}>1.20\times {\mathrm{Conflict}}^{R}$	High throttle demand while steering load remains high.
	LATE_THROTTLE_LOW_SPEED	${\mathrm{Smooth}}^{U}<0.80\times {\mathrm{Smooth}}^{R}\;\wedge \;V_{end}^{U}<V_{end}^{R}-1.0\;\mathrm{km}/h$	Hesitant throttle application, leading to poor exit speed.
Legacy	EXIT_SPEED_LEGACY	$\Delta V_{start}<-2.0\;\mathrm{km}/h\;\wedge \;P_{brake}^{max}<5\%$	Speed deficit inherited from the previous corner exit.

**Table 4:** Vehicle-and-track configurations used in the empirical case study.

Configuration	Vehicle	Track Layout
Setup 1	Mazda MX-5 Global Cup	Charlotte Motor Speedway Roval (2025)
Setup 2	Toyota GR86 Cup	Summit Point Raceway (Full Course)

**Table 5:** Sector designations for Charlotte Motor Speedway Roval and Summit Point Raceway.

Sector	Charlotte Roval 2025	Summit Point Raceway
1	T1 (Heartlands)	Main Straight and Braking
2	T2	T1 Right-hander
3	T3 (Infield)	T3 Left-hander
4	T4	T4 The Chute
5	T5/T6 Transition	T5 Carousel Entry
6	Hairpin Setup	T6/T7 Carousel Mid
7	New Hairpin	T8 Carousel Exit
8	Oval Turn 1	T9 The Bridge
9	Oval Turn 2	T10 Final Turn
10	Backstretch	Frontstretch
11	Bus Stop Entry	—
12	Bus Stop Apex	—
13	Bus Stop Exit	—
14	Oval Turn 3	—
15	Oval Turn 4	—
16	Final Chicane Entry	—
17	Final Chicane Exit	—
18	Frontstretch	—

**Table 6:** Reference lap-time baseline values for performance evaluation.

Track	Pilot	Reference Role	Lap Time
Charlotte Roval	Driver A	Expert (Ref.)	01:35.483
	Driver B	Intermediate (Ref.)	01:36.733
Summit Point	Driver A	Expert (Ref.)	01:18.983
	Driver B	Intermediate (Ref.)	01:19.767

**Table 7:** Lap-time performance comparison across evaluated circuits.

Track	Driver	Experience Level	Lap Time	Delta (s)	Delta (%)
Charlotte Roval	Driver A	Expert (Ref.)	01:35.483	–	–
	Driver B	Intermediate	01:36.467	+0.983 s	+1.03%
	Driver C	Beginner	01:37.917	+1.183 s ^a^	+1.22%
	Driver F	Beginner	01:41.283	+4.550 s ^a^	+4.72%
	Driver E	Beginner	01:43.833	+7.100 s ^a^	+7.36%
	Driver D	Beginner	01:47.883	+11.150 s ^a^	+11.56%
Summit Point	Driver A	Expert (Ref.)	01:18.983	–	–
	Driver B	Intermediate	01:20.583	+1.600 s	+2.03%
	Driver C	Beginner	01:22.100	+2.330 s ^a^	+2.89%
	Driver D	Beginner	01:22.583	+2.820 s ^a^	+3.50%
	Driver F	Beginner	01:22.633	+2.870 s ^a^	+3.56%
	Driver E	Beginner	01:23.333	+3.570 s ^a^	+4.43%

^a^ Calculated relative to the intermediate reference stint, Driver B.

**Table 8:** Segment classification audit. Segments are left unclassified when they contain fewer than 120 samples (2 s at 60 Hz) or when the signal is near-constant within the sector.

Channel	Segments	Classified	Short-Sectors	Near-Constant	Classified (%)
Brake (%)	4574	2378	146	2050	52.0
Combined G-Force (G)	4574	4428	146	0	96.8
Steering Angle (rad)	4574	4428	146	0	96.8
Throttle (%)	4574	3232	146	1196	70.7
All channels	18,296	14,466	584	3246	79.1

**Table 9:** Representative coaching output for Driver B vs. Driver A at Charlotte Roval. Sectors are sorted by descending time loss.

Sector	Corner	Δt (s)	Heuristics	LLM Coaching Output
12	Bus Stop Apex	+0.354	B3_LOW_BRAKE_EFFICIENCY B3_OVER_BRAKING_PEAK	Inefficient braking: too much brake pressure for low deceleration. Check for wheel lock or ABS overuse. Excessive peak braking: you are dropping the anchor too much.
7	New Hairpin	+0.324	R3_LATE_ROTATION B3_LOW_BRAKE_EFFICIENCY	Late rotation: you reached peak rotation after the corner apex. Try rotating the car earlier on entry. Inefficient braking: check for ABS overuse.

**Table 10:** Pre- and post-feedback fastest valid lap times.

Track	Driver	Pre	Δ Pre	Post	Δ Post	Gain/Loss	
		(mm:ss:ms)	(s)	(mm:ss:ms)	(s)	(s)	
Charlotte Roval	Driver A	01:35:483	—	—	—	—	—
	Driver B	01:36:467	+0.983	01:36:767	+1.283	+0.300	↑
	Driver C	01:37:917	+1.183	01:37:717	+0.983	−0.200	↓
	Driver D	01:47:883	+11.150	01:41:467	+4.733	−6.417	↓
	Driver E	01:43:833	+7.100	01:40:083	+3.350	−3.750	↓
	Driver F	01:41:283	+4.550	01:40:317	+3.583	−0.967	↓
Summit Point	Driver A	01:18:983	—	—	—	—	—
	Driver B	01:20:583	+1.600	01:19:717	+0.733	−0.867	↓
	Driver C	01:22:100	+2.333	01:21:117	+1.350	−0.983	↓
	Driver D	01:22:583	+2.817	01:20:717	+0.950	−1.867	↓
	Driver E	01:23:333	+3.567	01:22:867	+3.100	−0.467	↓
	Driver F	01:22:633	+2.867	01:22:233	+2.467	−0.400	↓

**Table 11:** Summary of post-feedback stochastic and performance evolution, Stints 1–5.

Driver	JSD Trend	FDR-Sig. ΔPE Channel	Throttle ΔPE	Lap-Time Gap
B (Intermediate)	Stable	Steering (Charlotte, S4)	n/a	+0.983→+1.283 s
C (Amateur, coached)	Decreased	Steering (S1–5, both)	>0	−42.1% (Summit)
D (Amateur, coached)	Decreased	Steering (S1–3, S5)	>0	−66.3% (Summit)
E (Amateur, uncoached)	Unchanged	None	>0	Decreased
F (Amateur, coached)	Decreased	Steering (S1–5, both)	>0 (highest)	−14.0% (Summit)

S*n* = Stint *n*; “both” = both circuits. Lap-time gap: change relative to the proficiency-matched reference; sign indicates increase (+) or decrease (−).

## Data Availability

The data used in this study is available on Github https://github.com/conect2ai/Racing4all.

## Abbreviations

The following abbreviations are used in this manuscript:

ABS	Anti-lock Braking System
ACC	Assetto Corsa Competizione
ADF	Augmented Dickey–Fuller
AI	Artificial Intelligence
BCa	Bias-Corrected and Accelerated
CI	Confidence Interval
DRL	Deep Reinforcement Learning
FDR	False Discovery Rate
HL	Hodges–Lehmann
Hz	Hertz
ICC	Intraclass Correlation Coefficient
JSD	Jensen–Shannon Divergence
JSON	JavaScript Object Notation
KL	Kullback–Leibler Divergence
KPSS	Kwiatkowski–Phillips–Schmidt–Shin
LLM	Large Language Model
PE	Permutation Entropy
PPO	Proximal Policy Optimization
RMS	Root Mean Square
SHAP	Shapley Additive exPlanations
SWE	Steering Wheel Entropy
UDP	User Datagram Protocol
WSS	Wide-Sense Stationarity
